# Silencing Is Noisy: Population and Cell Level Noise in Telomere-Adjacent Genes Is Dependent on Telomere Position and Sir2

**DOI:** 10.1371/journal.pgen.1004436

**Published:** 2014-07-24

**Authors:** Matthew Z. Anderson, Aleeza C. Gerstein, Lauren Wigen, Joshua A. Baller, Judith Berman

**Affiliations:** 1 Department of Genetics, Cell Biology and Development, University of Minnesota – Twin Cities, Minneapolis, Minnesota, United States of America; 2 Department of Microbiology and Biotechnology, George S. Wise Faculty of Life Sciences, Tel Aviv University, Ramat Aviv, Israel; The University of North Carolina at Chapel Hill, United States of America

## Abstract

Cell-to-cell gene expression noise is thought to be an important mechanism for generating phenotypic diversity. Furthermore, telomeric regions are major sites for gene amplification, which is thought to drive genetic diversity. Here we found that individual subtelomeric *TLO* genes exhibit increased variation in transcript and protein levels at both the cell-to-cell level as well as at the population-level. The cell-to-cell variation, termed Telomere-Adjacent Gene Expression Noise (TAGEN) was largely intrinsic noise and was dependent upon genome position: noise was reduced when a *TLO* gene was expressed at an ectopic internal locus and noise was elevated when a non-telomeric gene was expressed at a telomere-adjacent locus. This position-dependent TAGEN also was dependent on Sir2p, an NAD^+^-dependent histone deacetylase. Finally, we found that telomere silencing and TAGEN are tightly linked and regulated in *cis*: selection for either silencing or activation of a *TLO*-adjacent *URA3* gene resulted in reduced noise at the neighboring *TLO* but not at other *TLO* genes. This provides experimental support to computational predictions that the ability to shift between silent and active chromatin states has a major effect on cell-to-cell noise. Furthermore, it demonstrates that these shifts affect the degree of expression variation at each telomere individually.

## Author Summary

Genetic diversity is often high at telomeres, the chromosome ends where genes are readily amplified and modified. Phenotypic diversity, e.g., growth properties under a given condition, is affected by stochastic variations in gene expression exhibited among cells in a homogenous environment. Our studies found that individual subtelomeric genes show high variability of gene expression both between cells within a single population and also between separate sub-populations. Cell-to-cell variation, termed Telomere-Adjacent Gene Expression Noise (TAGEN), affected single telomeric genes. We found that classical telomeric silencing and TAGEN are tightly linked, with both being dependent upon proximity to telomeres and the Sir2 chromatin modifying enzyme. In addition, both are coordinately regulated locally—at the DNA level: at a telomere with transcription that is continually silenced or activated, the level of expression variability is reduced. This work provides experimental support for computational work that predicted this relationship between stochastic chromatin silencing and expression plasticity at each telomere individually. Furthermore, it demonstrates that these shifts affect the degree of cell-to cell noise of telomere-adjacent loci.

## Introduction

Responsiveness to minor changes in the environment requires exquisitely sensitive phenotypic plasticity. This can be executed via many different mechanisms, operating on different time scales, with different types of condition-specific responses, but usually includes changes in transcriptional and translational profiles. Variation between independent populations of cells that are presumed to be isogenic can be due to altered epigenetic properties, such as chromatin status of specific genes or chromosomal regions [1], [2], to cell-to-cell variations in gene expression [3], [4]. Such population and cellular variations are likely to operate continuously in natural environments. Microbes living within a mammalian host encounter a variety of host niches. For example, organisms that reside throughout the GI tract must be able to survive conditions in the oral cavity (pH 6.5–6.9, 33–35°C), the stomach (pH 2, 37°C), the small intestine (pH 7.4, 37–40°C), and anaerobic niches in the colon. Accordingly, the ability to acclimate rapidly to changing environments is thought to provide a selective advantage and is supported by studies in yeast and bacteria [5]–[9].

Gene expression noise, defined as cell-to-cell variation in levels of transcription and/or translation, provides phenotypic diversity within an isogenic population, enabling sister cells to respond differently to environmental challenges. Noise can be extrinsic, generally assumed to be due to differences in an environment or to natural variations in cell components such as transcription or translation factors that affect multiple alleles similarly [2], [3], [10]. By contrast, intrinsic noise is allele-specific and is often due to changes in the frequency with which transcription initiates from a given promoter [11], [12]. Intrinsic noise can provide a larger range of responses to environmental conditions, because the relative amounts of one gene product to another can shift more dramatically [13]. The quantitative contributions of extrinsic and intrinsic noise can be distinguished using different fluorescent protein fusions driven from otherwise identical alleles; extrinsic noise will result in correlated relative expression of both alleles, while intrinsic noise will result in independent relative expression of each allele [13]. The degree to which these types of noise contribute to different aspects of organismal survival by producing phenotypic diversity remains to be determined.

*C. albicans* is an organism that survives and flourishes in a wide range of niches within its human host. It engages in a benign commensal lifestyle, residing in the oral cavity and colonizing the GI tract [14]. In some hosts, especially following antibiotic treatment or immune suppression, it switches to a pathogenic state and becomes blood-borne, colonizing internal organs including the kidney, heart, or brain. *C. albicans* is generally found in the diploid state and it is known to tolerate high levels of genotypic and protein variation including aneuploidy and codon ambiguity [15]–[17]. Under stress conditions, e.g. during drug exposure, certain aneuploidies can provide improved fitness, largely due to increased expression of genes specifically found in extra copies on the aneuploid chromosomes [18]–[21]. Furthermore, while aneuploidy in general often incurs a high fitness cost, some aneuploidies have very little cost, even under non-selective conditions [22]–[24]. *C. albicans* also has a highly variable proteome because of the ambiguous CUG codon, which encodes serine most of the time. The CUG codons also encode leucine at low frequency in cells under non-stress conditions and at higher frequencies if cells are stressed [15].

*C. albicans* is the most virulent of the CUG clade organisms and this is thought to be due, at least in part, to amplification of several gene families thought to be important for virulence. These include the *SAP* [25], *LIP* [26], and *ALS* [27] gene families that encode proteases, lipases and cell wall adhesins, respectively. The most amplified of all the gene families in *C. albicans* are the *TLO* genes, present in 1 copy in most CUG family members, in 2 copies in *C. dubliniensis* [28] and in 14 copies in *C. albicans* [29]. All but one of the *TLO* genes is telomere-adjacent, usually found as the most telomere-proximal, or the penultimate, gene on the chromosome [30]. The *TLO* gene family encodes a set of related proteins with a Med2 domain, all of which are thought to function as exchangeable Med2 subunits for the Mediator transcription regulation complex [31]. However, how *TLO* gene expression is regulated and whether Tlo proteins contribute to the phenotypic plasticity of *C. albicans* has not been explored.

In many organisms, genes at telomeres are subject to telomere position effect (TPE), a transient transcriptional silencing due to specific chromatin complexes that are thought to nucleate at the telomeres and to spread inward along the chromosome arm [32], [33]. Studies of TPE generally detect two expression states (“ON” or “OFF”) using phenotypic read outs interpreted as indicating a biphasic open or closed chromatin state at a given telomere [34]. TPE is dependent upon the Silent Information Regulator proteins Sir2p, Sir3p and Sir4p in *S. cerevisiae* [35], [36]. Sir2p, an NAD^+^-dependent histone deacetylase (HDAC), is highly conserved in prokaryotes as well as eukaryotes [37] and contributes to silencing at the telomeres of organisms ranging from *S. pombe* to mice [38].

In *S. cerevisiae*, gene expression noise has been reported to be position-dependent. In one study, noise of two unrelated genes was shown to be influenced by their positions at internal loci on two different chromosome arms [12]. Bioinformatic meta-analysis of gene expression along all chromosome arms showed increased gene noise correlated with increased:1) proximity to the telomere; 2) prevalence of genes with promoters containing TATA box motifs; 3) intermediate levels of expression and 4) transitions between silencing-specific histone modifications [39]. The latter is not surprising, given that a number of histone modifiers affect gene expression noise through effects on transcription burst size as well as burst frequency [40]. This likely occurs through the regulation of nucleosome occupancy, which is different between promoters with TATA motifs and those without TATA motifs [41] and likely involves interactions with transcription factors as well [42].

Many of the chromatin modifier genes that affect noise encode HDACs. These include *RPD3* and *HDA1* [40]. In *C. albicans*, HDACs have been characterized to some degree, with Sir2 being reported to affect phenotypic switching under at least some conditions [43] and Hst3, Hda1, and seven other chromatin modifiers have been shown to alter white-opaque switching [44], [45]. Additionally, the Set3C complex, Set3 and Hos2, inhibit the yeast-to-filamentous transition by modulating transcriptional kinetics of key morphogenic regulators [46].

The association of noise with telomere proximity has only been explored experimentally in one study using *C. glabrata*, a pathogenic yeast most closely related to *S. cerevisiae*. *EPA1*, a subtelomeric gene that encodes a virulence-related adhesin [47], is subject to TPE and silencing contributed to high levels of *EPA1* gene expression noise [48]. This study detected effects at one telomeric locus but did not address the question of whether the effect was due to the telomere-adjacent position of the gene. Nonetheless, this work suggests that telomeric silencing by Sir2p may be associated with the highly variable expression of telomere-adjacent genes.

Here we investigated the expression of telomere-adjacent genes in *C. albicans*, with a focus on the *TLO* gene family. We detected high levels of variability between isogenic isolates at the population level, and, on average, genes that are most telomere-proximal on each chromosome have higher than average expression plasticity. Furthermore, telomere-adjacent genes exhibited high levels of noise (cell-to-cell variation in expression levels) that was largely due to intrinsic noise. Importantly, this telomere-adjacent gene expression noise (TAGEN) was dependent on genome position; *TLO* genes had lower noise levels when moved to an internal locus and a non-telomeric gene had higher noise when moved to a sub-telomeric locus. Similar to telomeric silencing, TAGEN was dependent upon NAD-dependent HDAC activity and, to a large degree, upon Sir2p. Finally, selection for either constitutive expression or constitutive silencing of a *TLO*-adjacent *URA3* gene specifically reduced the expression plasticity of the neighboring *TLO*, in *cis*, but had no effect on expression plasticity at other *TLO* genes in *trans*. Thus, TAGEN generates expression variability as a consequence of dynamic, local chromatin-mediated position-dependent silencing.

## Results

### Subtelomeric *TLO* transcript and protein levels are highly variable between different populations

**Figure 1:**
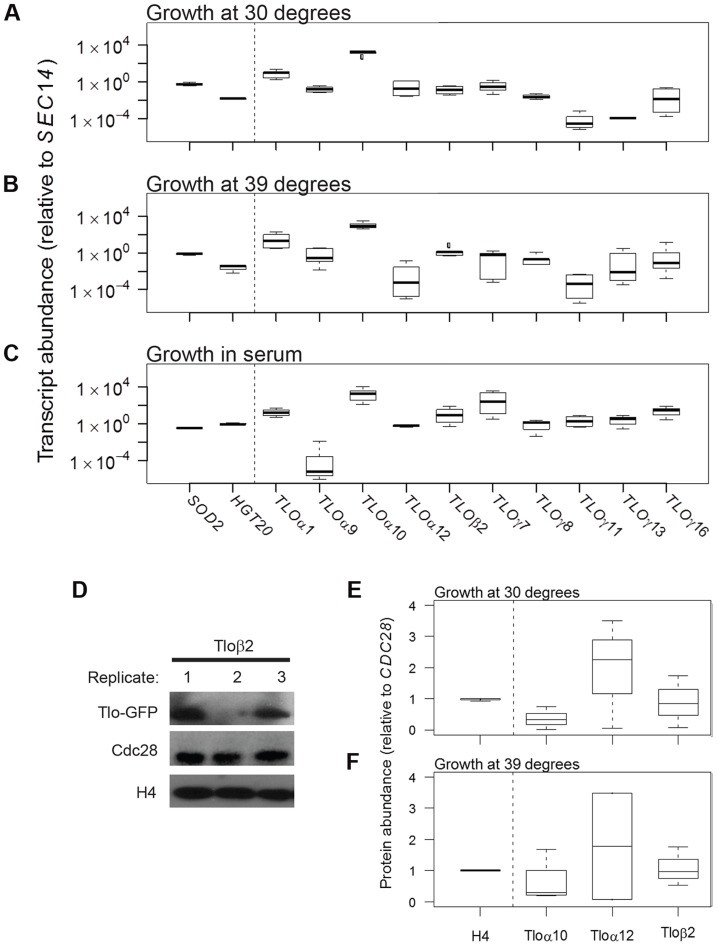
*TLO* expression is highly plastic at the transcript and protein level. qRT-PCR measured transcript abundance for ten *TLO*s representing all three clades in SC5314 and two control genes, *SOD2* and *HGT20*, that are expressed at similar levels. *TLO* abundance was measured for cells in logarithmic growth at (*A*) 30°C, (*B*) 39°C, and (*C*) under standard growth conditions supplemented with 10% serum. Transcript abundance was generally more variable for *TLOs* compared to control genes for all condition tested (variability indicated by the length of each box, which demarcates the first and third quartiles). (*D*) Protein abundance of Tlos and histone H4 was measured by Western blotting assay using Cdc28 as a loading control when cells were grown at either (*E*) 30°C or (*F*) 39°C. Tlo abundance was more variable compared to H4 in either condition regardless of clade. A Tloγ clade member, Tloγ5, was also similar variable but is expressed at much lower levels not on a similar scale to these proteins.

In the course of measuring *TLO* gene expression under a range of growth conditions, we found that expression levels for many individual *TLO* genes was strikingly variable (up to several orders of magnitude) between isogenic biological populations grown from single colonies under identical conditions (Fig. 1A–C). Furthermore, the level of *TLO* gene expression variation, measured as the coefficient of variation (CV; standard deviation divided by the mean; at least five replicates per gene-condition) [49], was far greater than that seen for two control genes, *SOD2* and *HGT20*, that were expressed at similar average levels, irrespective of the growth conditions (Table S4). Transcript abundance measurements were reproducible for individual populations (average standard deviation among technical replicates = 0.63 cycles vs. 4.43 cycles between biological replicates), further supporting the idea that the population-level expression of individual *TLO* genes varied considerably.

Genes with high cell-to-cell variation in gene expression are often differentially expressed across a large number of environments [41]. To ask if this is the case for the *TLO* genes, we analyzed an RNA-Seq dataset for expression of all *C. albicans* genes under eleven different environmental conditions [50]. Across growth conditions, the 13 telomeric *TLO* genes generally had high CV values relative to the average for all *C. albicans* genes analyzed by RNA-seq (Fig. S1A), and, as a group, their mean CV value was significantly higher than for a set of 13 randomly chosen genes (determined by examining 50,000 simulated gene sets, p<0.025, Fig. S1B). Cells either mock-treated or exposed to a variety of stresses were equally variable (Fig. S1C). Interestingly, the CV value for *TLOα34*, the only non-telomeric member of the *TLO* gene family, had a lower CV than the average telomere-adjacent *TLO* gene (Fig. S1A, blue arrow).

We next asked if Tlo protein levels were also variable. To detect individual Tlo protein levels, we constructed strains with a single copy of GFP fused to a given *TLO* gene and detected the fusion protein with an antibody to GFP. Tlo-GFP levels were highly variable among biological replicates grown from single colonies under identical conditions. For example, when different colonies expressing Tloβ2-GFP were prepared for protein extraction from independent log-phase cultures on the same day, the levels of GFP were much more different than a similar comparison of two control proteins (Fig. 1D). Two other Tlo-GFP fusion proteins (representing all three Tlo protein clades [51] showed similar variability when examined under several growth conditions (Fig. 1E, F). Of note, differences in the protein levels of Tlos generally were less dramatic as those seen for transcripts. Nonetheless, individual Tlo protein levels varied considerably between different biological replicate populations.

### *TLO* expression is less variable in more uniform environments

**Figure 2:**
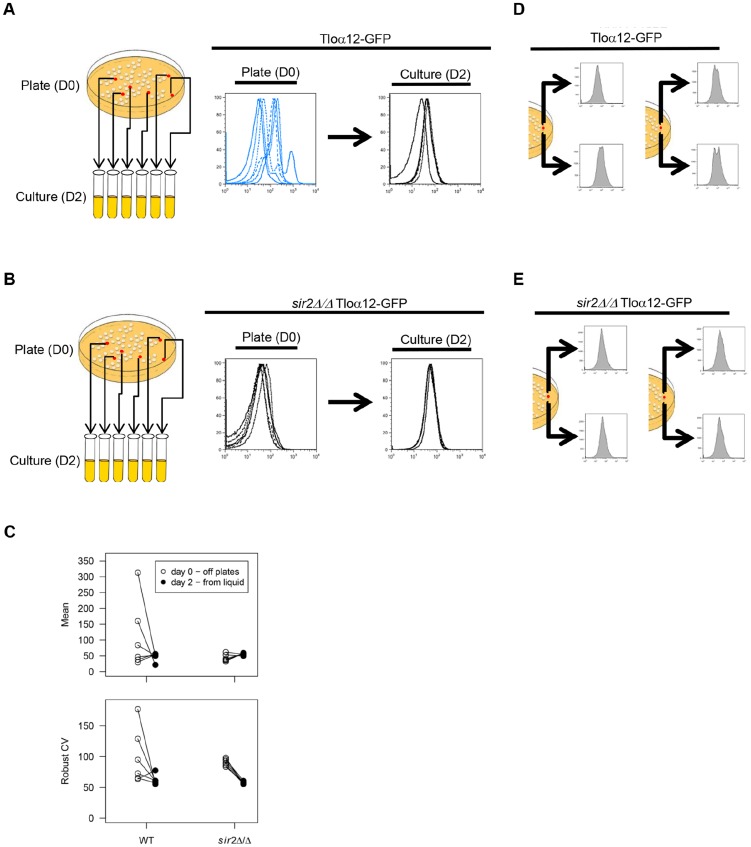
*TLO* noise and expression plasticity is greater in colonies than in liquid culture. Six Tloα12-GFP colonies in either a WT (*A*) or *sir2Δ/Δ* background (*B*) were picked from plates (D0) and passaged in liquid culture each day for two days (D2). Cells from these time points were fixed and analyzed by flow cytometry. (*C*) Flow cytometry profiles for Tloα12-GFP in the WT and *sir2Δ/Δ* background were analyzed for mean expression and robust CV for both the D0 and D2 time points and variability in both measures. Black lines connect the same cell population from D0 to D2. Variability in both mean expression and robust CV were reduced at D2 compared to D0 for both WT and *sir2Δ/Δ* backgrounds. Yet, Tloα12-GFP was always more variable in the WT than the *sir2Δ/Δ* background. Simultaneously, two regions of single Tloα12-GFP colonies were picked and assayed for fluorescence by flow cytometry in either a WT (*D*) or *sir2Δ/Δ* background (*E*). Fluorescence profiles of the two regions differed in the WT background but were much more similar when *SIR2* was deleted.

Expression variability between isolates could be the result of expression differences between whole populations or due to cell-to-cell variation within a population. We hypothesized that this high level of variability from population to population could be due to *TLO* gene expression differences originating from variability between colonies grown on solid agar plates. Based on the assumption that colony growth on solid media subjects cells to intense founder effects and/ot different local environments [52], [53], we asked if Tlo expression differences become less evident after cells from single colonies were propagated in liquid medium, assumed to be a more uniform environment that is also less sensitive to founder effects because cells are continuously mixed. To address this question, we compared Tloα12-GFP expression profiles from 6 individual colonies, originating from a single parent colony, that were grown on solid media plates and the same six populations after two days of passaging in a constantly agitated liquid medium (Fig. 2A). The irregular shapes of expression profiles for cells from individual colonies that were prepared for flow cytometry (by propagation in liquid medium for two hours), suggested that these cultures contained mixtures of different subpopulations. Furthermore, these profile shapes were different for the six colonies, suggesting different founder effects. Because cells lifted from a colony are closely related both genetically and epigenetically (more likely to be in the same silencing state), we think variability in silencing states and, potentially, the local environments within a colony produce these profile differences. In contrast, passaging the same colony isolates in liquid medium for two days resulted in expression profiles that were more regularly shaped and more similar to one another (Figure 2).

Passaging in liquid for two days did not significantly alter Tloα12-GFP mean expression (t_5_ = 1.38, p = 0.29) or mean robust CV (t_5_ = 1.90, p = 0.12) among the five wild-type populations. However, the variance among populations was significantly reduced for both mean expression (F_{5, 5}_ = 71.7, p = 0.0002) and robust CV (F_{5, 5}_ = 32.7, p = 0.002) (Fig. 2C). This suggests that either the populations became more homogeneous because distinct subpopulations were better mixed in liquid culture, and/or because Tloα12-GFP expression was more uniform in a more homogenous environment.

In *S. cerevisiae*, Sir chromatin modifiers affect telomeric silencing, with the Sir2p NAD^+^-dependent histone deacetylase (HDAC) being the most evolutionarily conserved. To ask if Sir2 regulates the colony-to-colony variation observed, we performed flow cytometry on different colonies expressing Tloα12-GFP in a *sir*2Δ/Δ strain. Mean fluorescence of Tloα12-GFP in a *sir*2Δ/Δ background did not change (t_5_ = −2.13, p = 0.087), while Robust CV significantly decreased (t_5_ = 14.01, p<0.0001) after liquid passaging (Fig. 2B, C). As in the wild-type background, both fluorescence intensity and Robust CV show less population-to-population variability after liquid passaging (mean fluorescence: F_{5, 5}_ = 10.93, p = 0.020; CV: F_{5, 5}_ = 8.76, p = 0.035). Comparing the variance among populations of wild-type and *sir*2Δ/Δ cells, the wild-type populations were always more variable than the *sir*2Δ/Δ populations, regardless of the parameter or the timepoint (D0, mean fluorescence: F_{5, 5}_ = 93.62, p = 0.0001; D2, mean fluorescence: F_{5, 5}_ = 14.27, p = 0.011; D0, CV: F_{5, 5}_ = 62.22, p = 0.0003; D2, CV: F_{5, 5}_ = 16.65, p = 0.0078). Thus, the absence of Sir2 protein reduced the founders effect seen in WT populations isolated from different colonies, suggesting that the function of wild-type Sir2 is to mediate the variation in expression of Tloα12-GFP.

To further test the founder effect on Tlo expression, we examined expression of Tloα12-GFP protein in cells originating from opposite sides of the same colony. Interestingly, flow cytometry profiles (after 2 hours of liquid growth) differed for the different colony regions (Fig. 2D), suggesting that populations of cells within a colony have different degrees of expression and that each population can have different levels of cell-to-cell noise. It also implies that the reduction in noise following overnight growth in liquid is not a simple function of more uniform mixing in the liquid media. Thus, it appears that colony regions have different levels of expression and of cell-to-cell noise (Fig. 2A, C, D). In contrast, flow cytometry profiles of Tloα12-GFP expression from different parts of a single *sir*2Δ/Δ colony were similar (Fig. 2E). Therefore, expression variability between and within single colonies is Sir2p-dependent. Furthermore, although microenvironments may differ within a colony [52], expression levels do not vary considerably within *sir*2Δ/Δ colonies, suggesting that the variation seen in wild-type cells is either not due to microenvironmental differences or that Sir2 is required to sense those microenvironmental differences. We propose that the variation at Tlo genes is primarily a function of intrinsic noise rather than a response to the microenvironment.

**Figure 3:**
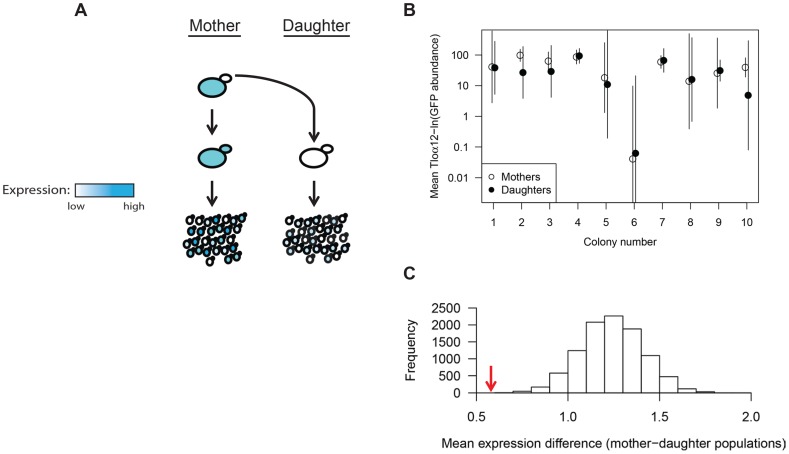
*TLO* expression state is heritable. (*A*) Ten Tloα12-GFP daughter cells were dissected away from their respective single mother cell. Both mother and daughter cells were grown independently for 18 hours and assayed for Tloα12-GFP expression by fluorescence microscopy of the resulting population. (*B*) Tloα12-GFP fluorescence of 50 cells for each population was collected by microscopy and the mean and standard deviation were plotted. Mother-daughter pairs were plotted together and generally show similar levels of mean expression between each pair, although more difference is evident in colonies 2 and 10 (*C*). The mean(ln) difference between cell expression data of mother-daughter pairs (red arrow) was tested against simulated datasets constructed from randomized mother-daughter affiliations(grey bars), and the association between mother-daughter pairs was highly significant.

To address the degree of heritability of Tloα12-GFP expression levels and expression noise, we analyzed the expression level of mother-daughter cell pairs by pedigree analysis. We isolated 10 mother-daughter pairs, dissected buds from mothers, and allowed them to grow separately on a plate for 18 hours (Fig. 3A). We compared populations of 50 cells from individual mothers to 50 cells from their own daughters to ask if these related populations were more similar to one another than expected by chance (Fig. 3B). The mean difference in absolute ln(expression) was 0.58 for the mother-daughter pairs and was 1.24 for randomized daughter pairs, with the 5% quantile at 0.96. Thus, the mother-daughter pairs were significantly similar to one another (p≤0.0001) than expected by chance (Figure 3C). Interestingly, two daughter populations (Colonies 2 and 10, Figure 3C) did not exhibit perfect overlap with their respective mother populations, indicating that expression similarity, although heritable, can diverge over a small number of generations.

### Tlo abundance varies from cell to cell

The studies above analyzed primarily variation in mean and CV of populations of cells. Gene expression noise is studied at the level of cell-to-cell differences, so we next measured cell-to-cell variation using fluorescence microscopy of individual cells isolated from multiple populations (originating from single colonies). We analyzed the cell-cell variation (measured as CV) within each population (founded from a single colony), and also compared the CV between different populations. For microscopy studies we analyzed 50 cells from each population of five Tloα and Tloβ clade fusion proteins, which localize to the nucleus and are expressed at higher levels (and thus are more detectable by fluorescence microscopy than Tloγ-clade genes) [51].

**Figure 4:**
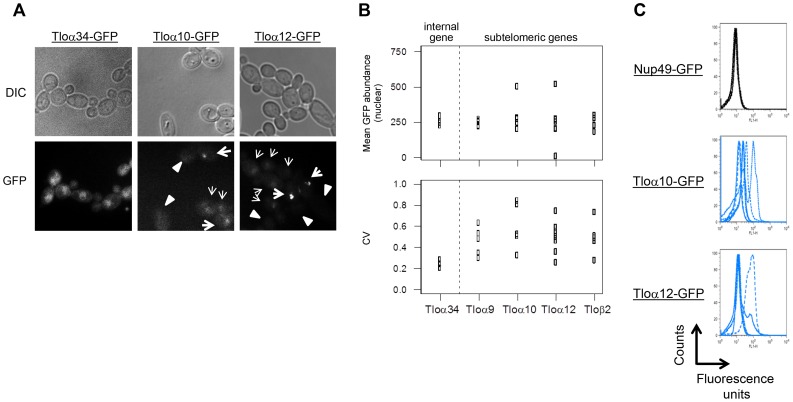
Subtelomeric *TLO*s exhibit cell-to-cell variance. (*A*) Tloα34, Tloα10, and Tloα12 tagged with GFP at the C-terminus were imaged to determine nuclear signal intensity of single cells. (*B*) Mean nuclear abundance of single cells GFP tagged at five Tlos was quantified using images as collected in (*A*). Mean GFP abundance of Tlos was similarly variable to the chromosome internal Tlo, Tloα34. However, variation of GFP abundance among single cells in a single replicate was greater for subtelomeric Tlos than Tloα34. At least four replicates were performed for each strain. (*C*) Flow cytometry profiles of Nup49, Tloα10, and Tloα12 tagged with GFP indicate the expression noise of cell within each population. An overlay of at least four experiments is shown.

Strikingly, the fluorescence signal for subtelomeric Tlo genes varied dramatically from cell-to-cell, ranging from very bright cells to cells with no obvious signal (Fig. 4A, B). The level of population-to-population variation was also higher for subtelomeric Tlo genes, consistent with the detection of expression plasticity at the population level (Fig. 1). Growth under stress conditions (5 mM H_2_O_2_ or cell wall stress) also resulted in high levels of Tloα12 cell-to-cell variation (Fig. S2; p<0.001; significance determined using a bootstrap procedure that compared the measured ratio of CV_Nup49-GFP_/CV_Tloα12-GFP_ against the critical value obtained from 10,000 simulated datasets that randomized the background of measured cells). Consistent with the RNA-seq results, the non-telomeric Tloα34-GFP gene, exhibited minimal cell-to-cell and population-to-population variation (Fig. 4A, B).

To measure gene expression levels for much larger numbers of cells, we analyzed GFP expression levels using flow cytometry (100,000 cells per population). Nup49, which encodes a nuclear pore component expressed at similar average levels to the Tloα and Tloβ proteins, exhibited minimal variation between cells within a population (evident by examining the peak width) and between populations (Fig. 4C, S3). In contrast, both cell-cell and population-population variability was much greater for Tlo-GFP than for Nup49-GFP fluorescence levels (Fig. 4C).

### Tlo cell-to-cell noise is intrinsic

**Figure 5:**
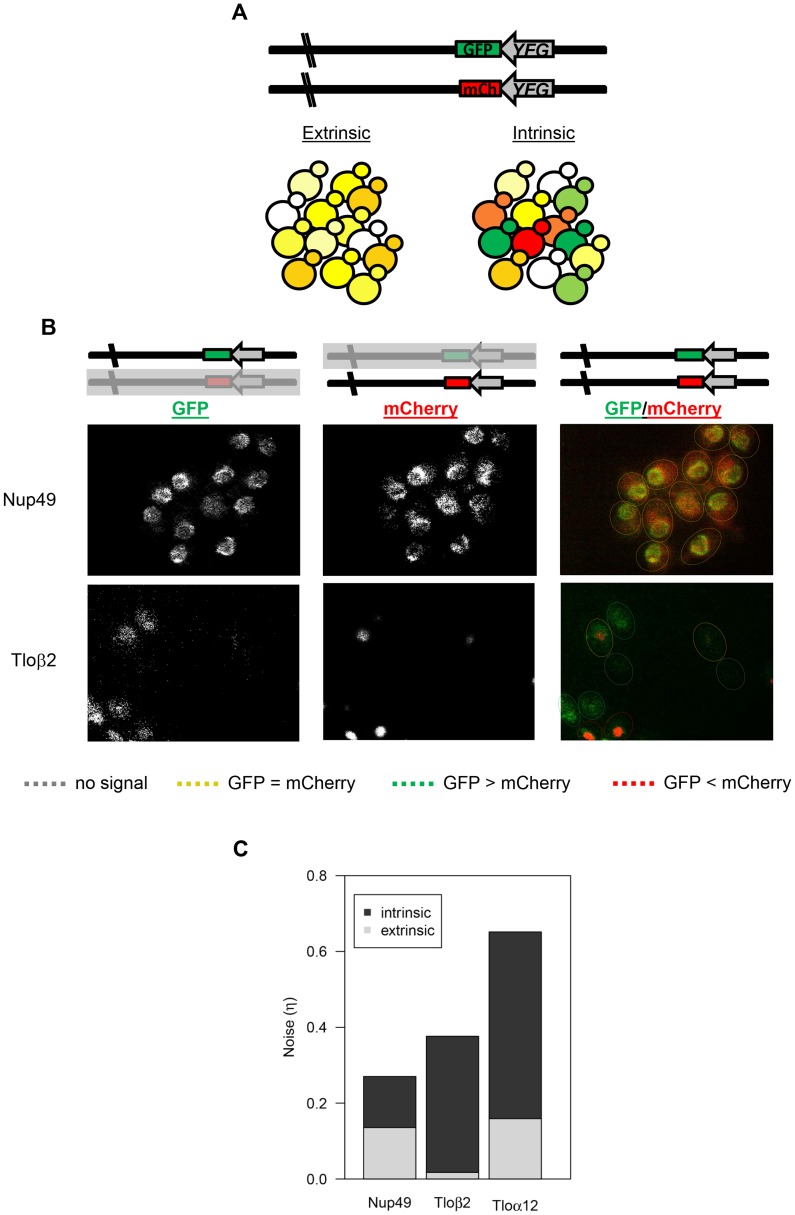
*TLO* noise has a large intrinsic component. (*A*) Schematic of the dual reporter system to identify intrinsic noise from expression of the two alleles for a single gene. Cells with the same amount of each tagged protein appear yellow, but cells expressing more of one fluorescent protein than the other appear green or red. (*B*) Relative GFP and mCherry abundance of tagged Nup49 and Tloβ2 is shown separately and as a merge. Cells are outlined to indicate similar or different levels of either fluorophore. Abundance of the GFP and mCherry-tagged alleles was similar for Nup49, indicating extrinsic noise. Tagged alleles of Tloβ2 exhibited a range of relative abundance and indicates significant intrinsic noise. (*C*) The intrinsic and extrinsic components to for Nup49, Tloα12, and Tloβ2 gene noise were calculated based on Elowitz et al, 2003. Both forms of noise contributed equally to Nup49 noise. However, intrinsic noise contributed to the majority of Tlo noise.

Two general sources of cell-to-cell variation have been explored extensively in many different species [1], [3], [12], [13], [40]. Extrinsic noise is due to conditions that differ between cells, such as a general level of ribosome or a local exposure to different growth conditions (Fig. 2). In contrast, intrinsic noise operates independently on different alleles of the same gene or promoter. The classic method to distinguish between extrinsic and intrinsic noise is to tag two different alleles of the same gene/promoter with two different fluorescent proteins and to observe the relative levels of each on a cell-by-cell basis. Accordingly, we tagged both alleles of *TLOα12* or *TLOβ2*, using GFP for one allele and mCherry for the other, and determined the degree to which each of the alleles was expressed in individual cells by fluorescence microscopy (Fig. 5A). Extrinsic noise manifests as variable yet correlated expression of the two alleles, while intrinsic noise results in independent, allele-specific expression levels.

The relationship between mCherry and GFP expression in Nup49 (control), Tloα12, and Tloβ2 were clearly different, based on fluorescence intensities (Figs. 5B, S4). In each individual population (12 populations for each tagged gene, see methods) a simple correlation test between the two fluorophores indicated that there were considerable differences for the three tagged genes (Fig. S4, Table S5). We considered the 12 populations for each gene as independent because different colonies and different locations within colonies were different enough from one another that they were not good predictors of the degree of either intrinsic or extrinsic noise (Table S5). The levels of both intrinsic and extrinsic noise (extrinsic: F_2, 33_ = 12.8, *p*<0.0001; intrinsic: F_2, 33_ = 26.5, *p*<0.0001, Fig. 5C) were different for the different genes measured. Post-hoc Tukey tests indicated the difference between the two types of noise; the two *TLO* genes both had significantly higher intrinsic noise than Nup49. On the other hand, extrinsic noise levels were not specific to *TLO* genes. Tloβ2 has significantly less extrinsic noise than Nup49 or Tloα12 (which were not different from each other). Furthermore, for both *TLO* genes, the contribution of intrinsic noise to total noise was significantly greater than the contribution of extrinsic noise (Tloβ2: t_11_ = −16.8, p<0.0001; Tloα12: t_11_ = −6.5, *p*<0.0001, Nup49 t_11_ = 0.056, p = 0.96, Fig. 5C).

### Subtelomeric position contributes to gene noise

To investigate whether increased expression plasticity is a general property of telomere-proximal genes, we examined the expression of sets of 16 genes starting with the most telomere-proximal and stepping sequentially into chromosome internal genes using the available *C. albicans* RNA-Seq dataset [50]. Both sets of the 16 most telomere-proximal genes (including 9 of 13 subtelomeric *TLO*s) and the set of 16 penultimate telomere-adjacent genes (including 4 of 13 subtelomeric *TLO*s) were significantly more transcriptionally variable than sets of 16 random genes (Fig. S5A, B; significance determined by a bootstrapping procedure as described above; *p*<0.025 in both cases). A similar trend was seen for the genes in the third-most telomere-proximal position (Fig. S5B). However, this pattern did not continue as a general trend along the chromosome (Figure S5C), indicating that any ‘spreading of TAGEN’ inwards from the telomere does not propagate more than ∼8 kb into the chromosome arms.

**Figure 6:**
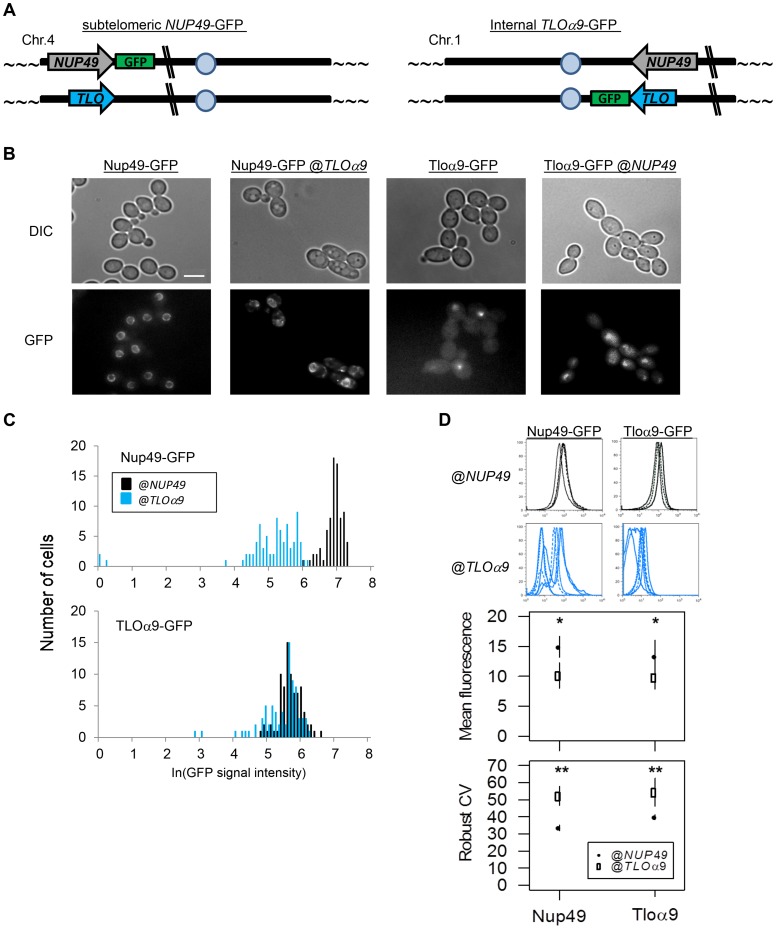
Gene noise and expression plasticity is elevated at the subtelomere in *C. albicans*. (*A*) A schematic identifies the ectopic location of subtelomeric Nup49-GFP and internal Tloα9-GFP in the gene position swap. (*B*) Fluorescence microscopy was performed for Nup49-GFP and Tloα-GFP at either the *NUP49* or *TLOα9* locus. GFP expression was stronger and more uniform for either gene at the *NUP49* locus compared to the subtelomeric *TLOα9* locus. (*C*) GFP expression from (*B*) was quantified for 100 cells from 2 biological replicates. Expression of either gene at the *NUP49* locus was higher than at the *TLOα9* locus. (*D*) Flow cytometry of Nup49-GFP and Tloα9-GFP also indicated reduced expression, increased expression plasticity, and increased noise at the subtelomeric *TLOα9* locus compared to the internal *NUP49* locus. * denotes p<0.05. ** denotes p<0.01.

Many studies of *S. cerevisiae* found that differences in promoter structure correlate with differences in the amplitude of gene noise [54], [55]. To determine the extent to which telomere position and promoter structure affect the variability of TLO gene expression, we constructed two *TLO-NUP49* swap strains (Fig. 6A): NUP49-GFP@TLO, in which the control gene *NUP49-GFP*, together with its native promoter, was moved to the sub-telomeric *TLOα9* locus on the left end of Chromosome 4 (YJB12963); and *TLOα9-GFP@NUP49*, in which *TLOα9*-GFP, together with its native promoter, was moved to the internal *NUP49* locus on the right arm of Chromosome 1 (YJB12966). Importantly, when either Nup49-GFP or Tloα9-GFP were expressed at the *NUP49* locus, noise (as measured by fluorescence microscopy) was significantly lower than when either of these proteins was expressed from the *TLOα9* locus (Fig. 6A–C, Fig. S6; *p*<0.05). Expression of Nup49-GFP and Tloα9-GFP was also significantly lower at the *TLOα9* locus compared to the *NUP49* locus (Fig. 6A–C; *NUP49:* t_85.42_ = 16.43, *p*<0.00001; *TLOα9:* t_85.44_ = 4.71, *p*<0.00001). Flow cytometric analysis of the four strains (two with tagged genes at their native loci and two with swapped loci) also indicated that genes at the subtelomeric *TLOα9* locus exhibit a significant decrease in the mean fluorescence signal (position: F_1_ = 5.04, *p* = 0.038, gene: F_1_ = 0.93, *p* = 0.35) and an increase in the level of gene noise (Robust CV; position: F_1_ = 10.12, *p* = 0.005, gene: F_1_ = 2.10, *p* = 0.17) relative to the internal *NUP49* locus (Fig. 6D). This suggests that the subtelomeric *TLOα9* locus is sufficient to cause increased noise because it is telomere-adjacent and affected by Telomere-Adjacent Gene Expression Noise (TAGEN), which influences both population-to-population (expression plasticity) and cell-to-cell (noise) variability. Furthermore, TAGEN appears to be independent of the promoters tested.

### *SIR2* affects TAGEN

**Figure 7:**
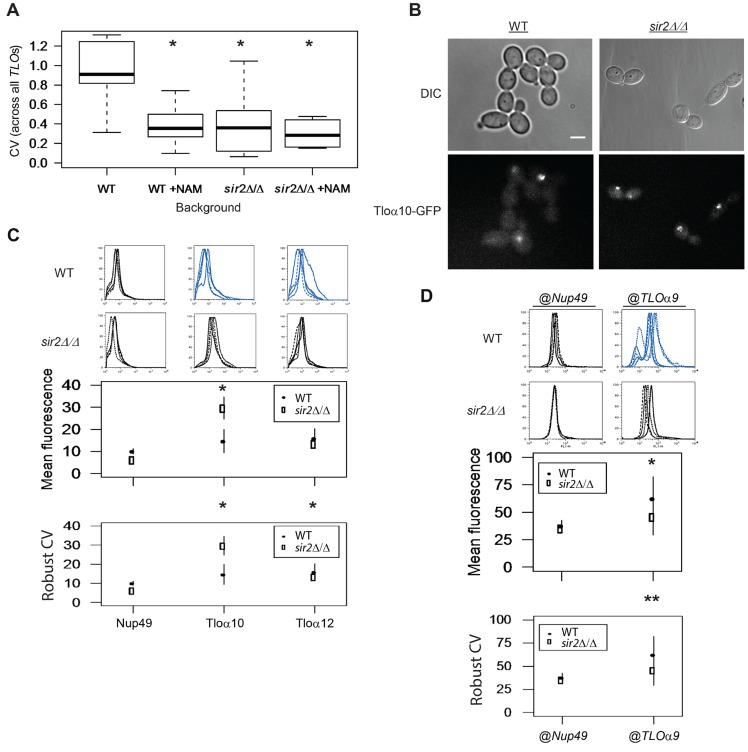
Sir2 contributes to *TLO* TAGEN. (*A*) Transcript abundance measurements of six *TLO*s and two control genes were collected from either *SIR2* or *sir2Δ/Δ* cells and in the presence or absence of the Sir-type HDAC inhibitor nicotinamide (NAM). Subtelomeric *TLO* expression plasticity specifically decreased when either treated with NAM or in the *sir2Δ/Δ* background but mean expression was not affected. Treatment of *sir2Δ/Δ* cells with NAM does not further decrease expression variability. (*B*) Fluorescence microscopy of GFP-tagged Tlos in either a *SIR2* or *sir2Δ/Δ* background showed reduced cell-to-cell variation in a *sir2Δ/Δ* background. (*C*) Flow cytometry of GFP tagged Nup49, Tloα10, and Tloα12 also identified significantly reduced noise for both Tlos in the *sir2Δ/Δ* background. Fluorescence signal of Tloα10 was also increased in a *SIR2* deletion strain. (*D*) Flow cytometry measured fluorescence signal of Nup49-GFP expressed at either the subtelomeric *TLOα9* or internal *NUP49* locus in both a *SIR2* and *sir2Δ/Δ* background. Gene noise of subtelomeric Nup49-GFP decreased significantly in the *sir2Δ/Δ* background. * denotes p<0.05. ** denotes p<0.01.

The Sir2p HDAC was required for *TLO* expression variability between colonies. Therefore, we hypothesized Sir2 may also influence *TLO* noise among cells in a single population. We first asked whether addition of nicotinamide (NAM), an inhibitor of NAD^+^-dependent HDACs, or deletion of *SIR2* had an effect on TAGEN at *TLO* genes using qRT-PCR. Addition of NAM or the lack of Sir2p significantly reduced expression plasticity (measured with qPCR, Fig. 7A, S7; background: F_1_ = 6.44, *p* = 0.020; NAM: F_1_ = 7.79, *p* = 0.011; interaction: F_1_ = 3.25, *p* = 0.086), while neither NAM nor the absence of Sir2p significantly influenced mean *TLO* gene expression (Fig. S7; Sir2 background: F_1_ = 0.03, *p* = 0.86; NAM: F_1_ = 1.42, *p* = 0.25). Furthermore, the effect of deleting *SIR2* together with NAM exposure affected expression and plasticity to a similar degree as either NAM or deletion of *SIR2* alone: reduced variability with little effect on expression levels (interaction; CV: F_1_ = 3.25, *p* = 0.086). Similar results for wild-type vs *sir*2Δ/Δ mutants were obtained by microscopy (Fig. 7B, S8; *p*<0.05) as well as by flow cytometry of Tloα10-GFP or Tloα12-GFP (Fig. 7C; Robust CV; gene: F_1_ = 1.21, *p* = 0.29; Sir2 background: F_1_ = 5.44, *p* = 0.03; interaction: F_1_ = 0.165, *p* = 0.69). Thus, Sir2p makes a significant contribution to expression plasticity of Tloα10-GFP and Tloα12-GFP.

To ask if Sir2p contributes to the position-dependent aspect of *TLO* TAGEN, we compared the level of expression noise for the Nup49-GFP@TLO *α9* locus in a *sir*2Δ/Δ strain relative to the level of expression noise for the Nup49-@TLO *α9* locus in a wild-type strain. Importantly, the expression noise for Nup49-GFP was decreased in a *sir*2Δ/Δ strain only for Nup49-GFP@TLO *α9* locus and not for Nup49-GFP at its native locus (Fig. 7D, Robust CV; position: F_1_ = 11.38, *p* = 0.005, background: F_1_ = 5.10, *p* = 0.042, interaction: F_1_ = 6.91, *p* = 0.021). Thus, the position-dependent and promoter-independent TAGEN seen at *TLO* genes is dependent upon Sir2p and, most likely, dependent upon its activity as a NAD^+^-dependent HDAC.

### Other chromatin modifiers contribute to *TLO* TAGEN

Telomeric silencing is considered to be a process by which telomeres toggle between “OPEN” and “CLOSED” chromatin states. Such a biphasic switch would be expected to generate two subpopulations of cells that would be distinguishable by flow cytometry as having different expression peaks. Yet, expression profiles of specific Tlo-GFP fusion proteins did not exhibit two clear peaks. This could be due to regulation of *TLO* expression by multiple factors [4] or a relatively fast rate of switching between two expression states [56]. Thus, we explored the role of additional chromatin modifiers in the regulation of *TLO* expression levels and the degree of *TLO* TAGEN. Nine modifiers were analyzed by qRT-PCR. *HST1* and *SET1* influenced expression plasticity (*HST1*: t_6_ = −2.89, *p* = 0.028, *SET1*: t_6_ = −2.60, *p* = 0.041) but not expression levels (*HST1*: t_6_ = −0.99, *p* = 0.36, *SET1*: t_6_ = 1.20, *p* = 0.27), while *HDA1*, *HOS2*, *HST2*, *PHO13*, *NAT4*, *RPD31*, and *SET3* had no effect on expression level or plasticity (Fig. S9 and data not shown). Consistent with the qRT-PCR results, deletion of *HST1*, a *SIR2* paralog that affects some telomere-associated genes in *S. cerevisiae* [57], [58], resulted in decreased fluorescence signal for two GFP-tagged Tlo proteins, Tloα10 (t_187.2_ = 7.03, *p*<0.0001) and Tloα12 (t_139.4_ = 5.30, p<0.0001) (Fig. S10A, B), as measured by fluorescence microscopy. Consistent with a role for Hst1 protein at internal as well as telomeric loci, the expression noise for Nup49-GFP at its native locus was reduced in the *hst1*Δ/Δ strain (p<0.05). Cell to cell noise in the *hst1*Δ/Δ strains was reduced at Tloα12 (p<0.01) but not at Tloα10 (Fig. S10A, C), relative to noise levels in the wild-type *HST1* parent strains. Thus, unlike Sir2p, which has a major position-dependent role in enhancing noise at telomere-adjacent loci, Hst1p affects expression noise at internal as well as telomere-proximal regions and it affects expression plasticity and noise of different *TLO* genes differently.

### Telomeric silencing and *TLO* expression plasticity are coupled in *cis*

**Figure 8:**
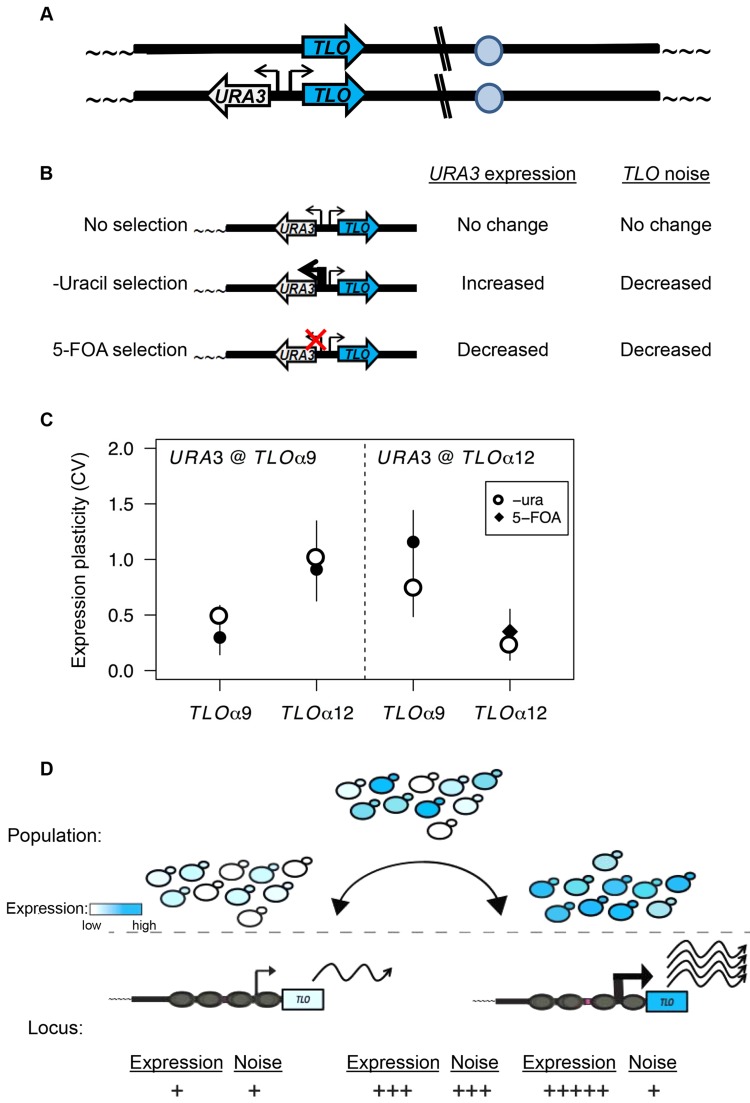
TPE produces *TLO* expression plasticity. (*A*) A cartoon represents *URA3* inserted into *TLO*-adjacent subtelomeres in a head-to-head orientation to test the effect of regulating *URA3* expression on *TLO* expression variability. (*B*). A diagram depicts the effect on *URA3* expression under growth in different conditions and the effect on *TLO* TAGEN. (*C*). qRT-PCR measured transcript abundance of *TLOα9* and *TLOα12* when *URA3* was either unselected, selected on media lacking uracil, or selected on 5-FOA. Selection of *URA3* expression significantly reduced expression plasticity of the adjacent *TLO* at either locus but not at the unlinked *TLO* gene. (*D*) Subtelomeric loci transition between active and inactive chromatin states. This transcriptional toggling results in a population of cells expressing subtelomeric loci over a wide range. Cells locked into a repressive transcriptional state have lower expression and reduced noise from transcriptional bursting at both the single cell and population level. Conversely, increased transcriptional activity, potentially due to loss of *SIR2*, increases expression and reduces noise due to increased transcriptional bursting.

We next asked if TAGEN and TPE are functionally related by measuring *TLO* expression variability in cells selected for constant expression or constant silencing of a *TLO*-adjacent selectable marker, *URA3*. We measured levels of the adjacent *TLO* (in *cis*) as well as an unlinked *TLO* (in *trans*), when cells were selected for expression of *URA3* (ON state selected on medium lacking uridine) or when cells were selected for repression of *URA3* (OFF state selected on medium containing 5-FOA) vs cells being free to ‘toggle’ between the two states (ON and OFF states, no selection on YPAD medium). We first constructed two strains, each with *URA3* inserted head-to-head at a *TLO*-adjacent position (adjacent to *TLOα9* or *TLOα12*; Fig. 8A) in the subtelomeres. These strains enabled the selection of cells expressing *URA3* (by growth in media lacking uracil (“-ura”)), or to select for silencing of *URA3* (by growth in the presence of 5-floroorotic acid (“5-FOA”)). Growth of *TLO-*adjacent *URA3* strains on media lacking uracil or with 5-FOA reduced or increased transcript abundance of *URA3*, respectively (data not shown). We then asked if selection in –ura or 5-FOA influenced *TLO* expression plasticity (Fig. 8B). Importantly, in both strains, selection either for or against *URA3* expression significantly reduced variability of the *URA3-*adjacent *TLO* transcript levels, yet it did not affect the transcript variability at an unlinked *TLO* (Fig. 8C; presence of selection: F_1, 20_ = 40.4, *p*<0.0001, gene: F_1, 20_ = 0.28, *p* = 0.60, interaction: F_1, 20_ = 0.174, *p* = 0.69). This occurred without a significant effect on expression levels (Fig. S11; presence of selection: F_1, 20_ = 0.03, = 0.87, gene: F_1, 20_ = 2.48, *p* = 0.13, interaction: F_1, 20_ = 0.145, *p* = 0.71). Thus, TAGEN at a specific *TLO* locus requires that cells toggle between the ON and OFF states and is lost if expression of an adjacent gene is constitutively ON or OFF. Furthermore, the effect of telomeric silencing on TAGEN occurs in *cis* and does not affect silencing or *TLO* expression at other subtelomeres.

## Discussion

Here, we discovered and characterized Telomere-Associated Gene Expression Noise (TAGEN), which is detectable not only as intrinsic variation at the cell-to-cell level but also generates variation at the population level. TAGEN is position-dependent, affecting only the most telomere-proximal genes, and it is reduced when cells are locked in a constant chromatin state or when cells are grown for multiple passages in liquid medium. TAGEN is subject to regulation by Sir2p in a position-dependent manner and also to other position-independent chromatin modifiers and transcription factors, e.g., Hst1p, which affect different *TLO* genes differently. Importantly, TAGEN is largely promoter-independent and it is tightly associated, in *cis*, with telomere position effect dynamics. Thus, TAGEN and TPE appear to reflect different aspects of the same phenomenon—the chromatin structure and its impact on gene expression at telomeres is dependent upon proximity to a telomere. Furthermore, increased expression plasticity and noise at telomere-adjacent genes (TAGEN) requires the dynamic process by which telomere-adjacent genes toggle between the ON and OFF states of expression presumably due to the OPEN and CLOSED states of telomeric chromatin.

At most genomic loci, noise is a phenomenon detectable only when cells are analyzed as individuals [13]. In contrast, TAGEN is detectable in populations of cells isolated from different colonies and also as a cell-to-cell variability largely due to intrinsic noise. The inherited epigenetic expression state is dependent upon telomere-adjacent position, *SIR2*, and the initial level of expression appears to exert a founder effect. Importantly, toggling or switching between the ON and OFF epigenetic state of cells in each population likely drives colony-to-colony variation seen at the population level (Fig. 8). A similar effect was seen for one telomere-adjacent gene, *EPA1*, in *C. glabrata* [48].

TAGEN is detected as large variations in levels of transcripts, measured by either qRT-PCR or by RNA-Seq (Figs. 1, S1). TAGEN is also evident at the individual cell level, when levels of GFP fusion proteins are measured by fluorescence microscopy or by flow cytometry (Figs. 2–7). This suggests that some of the transcriptional plasticity that affects *TLO* gene expression is buffered by post-transcriptional mechanisms, although we cannot rule out that the long half-life of GFP fusion proteins may contribute an additional buffering mechanism [59]. Since Tlo proteins are produced at levels far higher than they are needed [31], and since all *TLOs* encode a related subunit present in a single copy per Mediator complex, we suggest that excess Tlo proteins are likely subject to proteasome degradation [60]–[62].

Amplified gene families that promote growth within a relatively new environment are often located at telomeres. For example, *S. cerevisiae* strains used to produce wine, sherry or beer carry amplified *MEL*, *SUC*, and *MAL* genes, respectively, which promote breakdown of the predominant sugars in the respective fermentation processes. It is thought that the cost of amplification and diversification of gene family members is lower near telomeres [63]. In addition, the work here suggests that noise at telomeric loci may be exacerbated in a non-uniform environment (Fig. 2C). The fact that this noise is Sir2p-dependent suggests that it is a function of both TAGEN and TPE. Increased gene noise is also associated with duplicated genes [64], a common feature of expanded gene families at telomere ends. Based on this idea, subtelomeric loci populated with gene families would be expected to be transcriptionally noisy because of the reduced fitness costs associated with noise when multiple functional homologs are present. Bioinformatic analysis of gene expression in *S. cerevisiae* found that telomere-adjacent loci were expressed with higher levels of transcriptional noise [39].Thus, telomeres are not only safe neighborhoods for gene amplification but they are noisy neighborhoods for gene expression. We suggest that, because increased noise in non-uniform conditions is Sir2p-dependent, that it is intrinsic feature of TAGEN and, most likely, of TPE as well.

Intrinsic noise is generally thought to be influenced by the chromatin state at a given locus and is often ascribed to specific promoter structures or to interactions with specific components of the transcription regulation machinery. Consistent with this, most chromatin modifiers affect either the transcription burst frequency (frequency with which a promoter switches into a transcriptionally active state) and/or the transcription burst size (the total number of transcripts or proteins produced during each transcriptionally active state) [40], [56], [65]. Interestingly, mutations affecting TAGEN often reduced the noise level without causing a substantive change in gene expression levels (Figs. 7, S7, S9). We suggest that regulating the rate of switching between silent and active chromatin at telomeres will reduce the noise, even if it does not affect the net expression levels [3], [56]. Thus, TAGEN levels are dependent upon the frequency with which telomeric silencing opens and closes the chromatin.

TAGEN is dependent upon NAD^+^-dependent HDACs. Sir2p and the Sir2-like Hst1p contribute to TPE in *S. cerevisiae* as well as in *Schizosaccharomyces pombe*, *Plasmodium falciparum* and *Drosophila melanogaster* [66]–[68]. This provides further support for the idea that both processes are likely related to one another. TAGEN shows fairly smooth distributions of different expression levels per cell through a population (Figs. 2, 4, 6–7), yet TPE is considered a biphasic switch between two states [34], [69]. This is likely because TPE is often measured as a growth phenotype that must cross a specific threshold to be detected [33], [70] and has been considered as a largely population effect. In contrast, we measured TAGEN at the molecular level and, thus, detected a continuous distribution of expression levels and high levels of intrinsic noise. Importantly, the two processes appear to be inextricably linked: when cells with a *TLO*-adjacent *URA3* gene were selected for *URA3* expression to be either in all “OFF” or all “ON”, expression levels for the adjacent *TLO* gene were less variable than when no selective pressure was applied (Fig. 8D). This supports the idea that TAGEN is a consequence of dynamic switching between TPE states, rather than a consequence of silencing or depression of telomere gene expression per se.

In *C. albicans*, *TLOs* all encode the Med2 subunit of Mediator. In *S. cerevisiae*, Mediator interacts with Sir2 to modulate TPE [71], [72]. If a similar relationship exists in *C. albicans*, then one would expect Tlo proteins to be components of the silencing machinery itself. Consistent with this, a strain lacking Med3p, which interacts with Tlo proteins in the *C. albicans* Mediator complex tail, exhibits lower levels of TAGEN (data not shown). Thus, noisy *TLO* expression may contribute to TAGEN, and may proscribe an interesting feedback circuit. Whether the amplification of *TLO* genes has been an important adaptation for the recently evolved virulence features of *C. albicans*, and whether TAGEN and Mediator feedback play a role in this process remains to be determined.

## Materials and Methods

### Ethics statement

N/A

### Growth conditions used

Yeast cells were grown in standard conditions in rich medium (YPAD) at 30°C [73] unless noted otherwise. Assays were performed by diluting an overnight culture 1∶100 in fresh YPAD and grown at 30°C, 39°C, with 10% fetal bovine serum, with 5 mM H_2_O_2_, with 100 µg/µl Congo Red, or with 2 mM nicotinamide for 4 hours, as indicated.

### Strain construction

Strains are listed in Table S1. Transformations were performed using lithium acetate as previously described [73]. Strains carrying *NUP49* and *TLO* tagged with GFP or mCherry at the C-terminus were constructed by PCR amplification from plasmid p1602 [74], p2120, or p2343 [75], which contain GFP and *URA3*, GFP and *NAT1*, or mCherry and *NAT1*, respectively, using primers with at least 70 bp of homology to the target gene (Table S2). Correct insertion of the fluorescent protein in frame with the relevant *TLO* gene was first detected as described previously [51]. Only strains in which insertion was detected as a single unambiguous PCR fragment from a single chromosome arm were analyzed further. Integration of the construct at the expected locus was confirmed by PCR, Sanger sequencing, and Southern Blot analysis as described [51].

Locus swapping strains (Fig. 4) were constructed using a PCR amplicon containing the full open reading frame (ORF) to be moved, including either all sequences up to the adjacent open reading frames or 1 kb upstream and 1 kb downstream, whichever was shorter, the fluorescent tag, and the selectable marker from previously constructed strains. Transformation and screening were performed as described above.

### Quantitative reverse transcriptase-PCR (qRT-PCR) to measure *TLO* transcription

Transcript abundance measurements by qRT-PCR were performed as described [51] with primers listed in Table S3. Absolute quantification of SYBR fluorescence using the 2^nd^ derivative maximum value was used to calculate ΔCT values using *SEC14* as a control. All qRT-PCR results represent the average abundance of at least four independent cultures for each strain of interest.

### Analysis of Illumina Whole Transcriptome Shotgun Sequencing (RNA-Seq) data

RNA-Seq data for *C. albicans* grown under 11 different conditions in biological duplicates was obtained from Bruno *et al* [50]. We determined the coefficient of variation (CV = standard deviation divided by the mean) for each gene in each of the eleven environments that data were available for. We then averaged across all environments to determine the average CV for each gene. To determine whether a group of genes was significantly more transcriptionally variable than average, we conducted a bootstrap procedure to obtain a distribution of mean CV values for a group of genes of the appropriate size (i.e., 13 to examine *TLO* expression plasticity, 16 to examine position effects). We simulated 50 000 gene groups using the ‘sample’ function in the R Programming Language on the 6006 ORFs measured in the Bruno dataset; the 97.5% quantile of these 50,000 datasets was used to determine the critical value.

### Western blot of Tlo abundance

Protein lysates were collected as previously described [76]. Briefly, cells were inoculated into liquid YPAD cultures and grown overnight to stationary phase at 30°C with constant shaking. A 1∶100 dilution was then transferred to fresh YPAD and grown for four hours at 30°C with constant shaking prior to collecting lysates. Proteins were separated on a 12% polyacrylamide geland transferred to PVDF membrane (Immobilon-P, Millipore, Billerica, MA) as previously described [75]. Western blots were performed with mouse anti-GFP (Roche, Penzberg, Germany), rabbit anti-H4 (Santa Cruz Biotechnology, Santa Cruz, CA), and mouse anti-PSTAIR ab10345 (abcam, Cambridge, MA) followed by HRP-anti mouse or HRP-anti rabbit antibody (Santa Cruz Biotech, Santa Cruz, CA). Densitometry of band intensities was quantified using Fiji/ImageJ v1.46 (NIH, Washington D.C, District of Columbia).

### Mother-daughter cell dissection

*TLOα12-*GFP cells were struck onto SDC agar plates. Ten single cells were isolated using an Olympus BX40 dissecting microscope and followed during growth and division. Following the first division the mother and daughter cells were separated and allowed to grow up for 18 hours on the SDC agar plate. Tloα12-GFP expression was visualized by microscopy. We compared the mean difference in absolute ln(expression) values from colonies of daughter cells with 10,000 randomized affiliations.

### Fluorescence microscopy

Overnight cultures in YPAD were diluted 1∶100 in fresh SDC medium and grown at 30°C for 3–4 hours. DNA was stained with DAPI (4′,6-diamidino-2-phenylindole) (Sigma, St. Louis, MO) diluted 1∶1000 for 25 minutes, washed twice in fresh SDC, and imaged using differential interference contrast (DIC) and epifluorescence microscopy with a Nikon Eclipse E600 photomicroscope (Chroma Technology Corp., Brattleboro, VT). Digital images were collected using a CoolSnap HQ camera (Photometrics, Tucson, AZ) and MetaMorph software, version 6.2r5 (Universal Imaging Corp., Downingtown, PA). A total of 8 fields, were collected with 8 fluorescent images along the *z* axis, in 1-µm increments, for each cell to insure that any signal present was captured throughout the diameter of the cell. Exposure times were 500 ms for Nup49 and Tlo fluorescent fusion proteins. Projections of the *z* series were constructed with the stack arithmetic/sum function of MetaMorph for analysis and presentation.

Fluorescent-tagged protein abundance for each cell was measured by subtracting the average pixel intensity of three 4×4 regions of adjacent background from each of three 4×4 pixel regions within each nucleus. The signal intensity was defined as the average of the three background-subtracted nuclear regions. Nuclear signal intensity was determined for all cells in a minimum of 50 cells for each strain of interest. For all experiments, an equal number of cells were examined for expression and noise; for strains where data from more than the minimum number cells was collected, we used the ‘sample’ procedure in the R programming language [77] to randomly select cells to be analyzed.

Extrinsic and intrinsic noise was calculated as in Elowitz et. al [13]. Three strains (NUP49-GFP/NUP49-mCherry, TLOβ2-GFP/TLOβ2-mCherry, and TLOα12-GFP/TLOα12-mCherry) were streaked onto YPAD solid agar plates. Three colonies were chosen for each strain and cells from four regions of each colony were sampled (two from the edges of the colony and two from the center). These cells were suspended in liquid and the expression of the GFP and mCherry tagged genes was quantified by fluorescence microscopy for 50 cells using the method described above. The cells were also then cultured in liquid YPAD media for two days with passaging every 24 hours. Cells were taken in logarithmic growth (OD600∼0.5) after two days and 50 cells were measured again for GFP and mCherry fluorescence signal by microscopy.

### Flow cytometry of GFP-tagged strains

Cells for flow cytometry were prepared using a modified protocol from Sudbery [78]. An overnight culture in YPAD was diluted 1∶100 in fresh SDC media and grown at 30°C for 3–4 hours. Cultures in mid-logarithmic growth (OD600∼0.5) were collected at 1500×g, resuspended in 4% methanol-free formaldehyde (Thermo Scientific, Rockford, IL), and incubated on a tube rotator for 30 minutes. Cells were then spun down and resuspended in ice cold methanol for 3 minutes, washed three times in 55 mM HCl, resuspended in 500 µl of 5 mg/ml pepsin in 55 mM HCl, and incubated for 30 minutes at 37°C with gentle shaking. Cells were collected by centrifugation, washed three times with 1 ml of 10 mM Tris (pH 7.5), and resuspended in 460 µl Buffer A [78]. Cells were incubated in 40 µl of 1 mg/ml Zymolyase-20T (ICN Biomedicals, New York, New York) in 0.1 M phosphate buffer (pH 7.5) and 1 µl β-mercaptoethanol for 30 minutes at 37°C with gentle shaking and washed 5 times with 1% bovine serum albumin (BSA) in phosphate-buffered saline (PBS). Cells were resuspended in 500 µl of primary antibody polyclonal anti-GFP, ab290 (abcam, Cambridge, UK) diluted 1∶1000 in 1% BSA in PBS, and incubated overnight on a rotisserie at 4°C, washed 5 times in PBS. Secondary antibody (500 µl Alexa Fluor 488 donkey anti-rabbit (Invitrogen, Carlsbad, CA) diluted 1∶2000 in 1% BSA in PBS) was added, samples were incubated 45 minutes in the dark, cells were washed 5 times with PBS, resuspended in 1 ml PBS, and sonicated at 20% duty cycle three times.

Flow cytometry was performed using a FACSCalibur (BD Biosciences, San Jose, CA). Measurements were collected for 100,000 events and analyzed using FlowJo (Ashland, OR). Events were initially examined on a plot of SSC by FSC and gated to include all events (cells) that had measurable FSC and SSC. Mean expression and the Robust CV (100*0.5*(Intensity [at 84.13 percentile] – Intensity [at 15.87 percentile])/Median) of the gated population were collected using cell fluorescence measurements from the FL1 (fluorescein/GFP) channel. These measurements were the basis for further analysis.

### Silencing of *TLO*-adjacent *URA3*

Subtelomeric *URA3* was inserted in a head-to-head orientation immediately upstream of the *TLO* promoter (∼600 bp upstream of the *TLO* start codon) to produce a subtelomeric, *TLO*-adjacent *URA3*. Insertion sites were identified by PCR and sequencing as well as separation of chromosomes on contour-clamped homogenous electric field (CHEF) karyotype gens and Southern blotting.

Strains containing a *TLO*-adjacent *URA3* were grown in liquid YPAD and plated for single colonies onto YPAD for no selection of *URA3* expression, synthetic complete media (SDC) lacking uracil to select for *URA3* expression, and 5-floroorotic acid (5-FOA) to select for *URA3* silencing [70]. Five colonies from each condition for three different experiments were assayed for gene expression by qRT-PCR as described above.

## Supporting Information

Figure S1 Elevated *TLO* transcriptional variation identified by RNA-seq. (*A*) The CV of all genes assayed by RNA-seq in Bruno, et al, was calculated averaging across all eleven conditions. The CV of the thirteen expressed *TLO*s is indicated by red arrows. (*B*) The average CV of TLO genes (red arrow) was compared against the CV from 50000 simulated datasets of 13 random genes. The 95% quantile of these datasets is indicated with the vertical dashed line. (*C*) The CV of each *TLO* (“TLO”) and all genes expressed within two standard deviations of *TLO* genes by RNA-seq (“non-*TLO*”) was plotted for each condition tested in Bruno, et al.
(TIF)

Figure S2 *TLO*s exhibit cell-to-cell variance under stress conditions. (*A*) GFP expression was quantified by microscopy for 150 cells from 3 biological replicates each of Nup49-GFP and Tloα12-GFP strains. The mean expression and CV were plotted for each replicate. (*B*) GFP expression of individual cells from (*A*) was quantified. (*C*) The ratio of Tloα12-GFP to Nup49-GFP CV was tested against simulated datasets constructed from all expression data for a single condition. GFP abundance was significantly more variable for Tloα12-GFP compared to Nup49-GFP.
(TIF)

Figure S3 Variability in Nup49-GFP populations is low. Protein abundance of Nup49 and histone H4 was assessed by Western blot assay using Cdc28 as a loading control. Nup49 expression was similar among all three biological replicates.
(TIF)

Figure S4 Subtelomeric *TLO*s have elevated intrinsic noise. Abundance of GFP and mCherry signal for single cells in Nup49, Tloβ2, and Tloα12-tagged cells was plotted for at least 50 cells from twelve biological replicates, four regions of three separate colonies. A best fit line and the intrinsic and extrinsic components of noise were calculated for each sample. Intrinsic noise was significantly greater for Tloα12 and TLOβ2 than for Nup49.
(TIF)

Figure S5 Expression plasticity is associated with telomere proximity. (*A*) The CV between replicates for the five most telomeric genes from each chromosome arm was averaged across all eleven condition tested in Bruno, et al. The CV of each telomere adjacent gene was compiled based on position and plotted from the most telomeric gene “T” to the most centromeric gene “T-5”. The red diamond indicates the mean CV for that position. (*B*) The average CV of the 16 most telomeric genes (“T”, blue arrow), 16 telomeric-1 genes (“T-1”, purple arrow), and 16 telomeric+2 genes (“T-2”, green arrow) was compared against the CV from 50000 simulated datasets of 16 random genes. (*C*) The average CV for all genes was plotted against their genomic position. A small but significant decrease in noise was identified with increased distance from the centromere.
(TIF)

Figure S6 Gene noise increases at the subtelomere. Significance of the coefficient of variation ratio between expression of either gene at the *TLOα9* and *NUP49* locus was tested compared to randomized assignment of gene expression at the two loci. The noise ratio of the collected expression data was beyond the critical value (dashed line) indicating significantly elevated noise at the subtelomeric *TLOα9* locus.
(TIF)

Figure S7 Gene expression plasticity of individual *TLO*s is affected by Sir-type HDAC function. The transcript abundance and CV of two control genes, *SOD2* and *HGT20*, and six subtelomeric *TLO*s was plotted from either *SIR2* or *sir2*Δ/Δ cells and in the presence or absence of nicotinamide.
(TIF)

Figure S8 *SIR2* contributes to Tlo noise. GFP expression was quantified by microscopy as shown in Figure 6B for 78 cells from 2 biological replicates (*A*) and the ratio of the CV in the WT to the *sir2Δ/Δ* background was tested against simulated datasets (B) constructed from all expression data for a single gene in either background. Analysis of the expression data identified significantly reduced noise for Tloα10 and Tloα12 associated with deletion of SIR2 but not for Nup49.
(TIF)

Figure S9 Expression plasticity of individual *TLO*s is affected by additional chromatin modifiers. The (*A*) transcript abundance and CV of two control genes and six subtelomeric *TLO*s was plotted from either WT, *rpd3*Δ/Δ, *hda1*Δ/Δ, *hst1*Δ/Δ, or *set1*Δ/Δ cells. (*B*)Expression variability of *TLO*s was significantly reduced by deletion of *HST1* and *SET1*.
(TIF)

Figure S10 Hst1 and Set1 influence gene noise. (*A–C*) Fluorescence microscopy (*A*) analysis of GFP-tagged Tlos and Nup49 was performed in either a *HST1* or *hst1*Δ/Δ background. GFP expression was quantified (*B*) for 100 cells from 2 biological replicates and the ratio of the CV in the WT to the *hst1*Δ/Δ background was tested against simulated datasets (*C*) constructed from all expression data for a single gene in either background. Analysis of the expression data identified significantly reduced fluorescence signal for both Tlos and reduced noise for Tloα12 but not Tloα10 in the *hst1*Δ/Δ background. Noise was also reduced for Nup49 in the *hst1*Δ/Δ background.
(TIF)

Figure S11 TAGEN does not alter *TLO* expression. qRT-PCR measured transcript abundance of *TLOα9* and *TLOα12* when *URA3* was either unselected, selected on media lacking uracil, or selected on media containing 5-FOA. Selection of *URA3* expression did not significantly alter expression of either the adjacent or unlinked *TLO* gene.
(TIF)

Table S1 *C. albicans* strains used in this study.
(TIF)

Table S2 Primers used for strain construction.
(TIF)

Table S3 Primers used for *TLO* quantitative PCR.
(TIF)

Table S4 Gene expression coefficient of variation (CV) from qRT-PCR.
(TIF)

Table S5 Correlation of GFP and mCherry fluorescence in tagged strains.
(TIF)

## References

[1] Bar-Even A, Paulsson J, Maheshri N, Carmi M, O'Shea E, et al (2006) Noise in protein expression scales with natural protein abundance. Nat Genet 38: 636–643. 16715097 10.1038/ng1807

[2] Newman JR, Ghaemmaghami S, Ihmels J, Breslow DK, Noble M, et al (2006) Single-cell proteomic analysis of S. cerevisiae reveals the architecture of biological noise. Nature 441: 840–846. 16699522 10.1038/nature04785

[3] Raser JM, O'Shea EK (2005) Noise in gene expression: origins, consequences, and control. Science 309: 2010–2013. 16179466 10.1126/science.1105891 PMC1360161

[4] Octavio LM, Gedeon K, Maheshri N (2009) Epigenetic and conventional regulation is distributed among activators of FLO11 allowing tuning of population-level heterogeneity in its expression. PLoS Genet 5: e1000673. 19798446 10.1371/journal.pgen.1000673 PMC2745563

[5] Hernday AD, Braaten BA, Broitman-Maduro G, Engelberts P, Low DA (2004) Regulation of the pap epigenetic switch by CpxAR: phosphorylated CpxR inhibits transition to the phase ON state by competition with Lrp. Mol Cell 16: 537–547. 15546614 10.1016/j.molcel.2004.10.020

[6] Maamar H, Raj A, Dubnau D (2007) Noise in gene expression determines cell fate in Bacillus subtilis. Science 317: 526–529. 17569828 10.1126/science.1140818 PMC3828679

[7] Sumner ER, Avery SV (2002) Phenotypic heterogeneity: differential stress resistance among individual cells of the yeast Saccharomyces cerevisiae. Microbiology 148: 345–351. 11832498 10.1099/00221287-148-2-345

[8] Acar M, Mettetal JT, van Oudenaarden A (2008) Stochastic switching as a survival strategy in fluctuating environments. Nat Genet 40: 471–475. 18362885 10.1038/ng.110

[9] Levy SF, Ziv N, Siegal ML (2012) Bet hedging in yeast by heterogeneous, age-correlated expression of a stress protectant. PLoS Biol 10: e1001325. 22589700 10.1371/journal.pbio.1001325 PMC3348152

[10] Luca AC, Mersch S, Deenen R, Schmidt S, Messner I, et al (2013) Impact of the 3D microenvironment on phenotype, gene expression, and EGFR inhibition of colorectal cancer cell lines. PLoS One 8: e59689. 23555746 10.1371/journal.pone.0059689 PMC3608563

[11] Eldar A, Elowitz MB (2010) Functional roles for noise in genetic circuits. Nature 467: 167–173. 20829787 10.1038/nature09326 PMC4100692

[12] Becskei A, Kaufmann BB, van Oudenaarden A (2005) Contributions of low molecule number and chromosomal positioning to stochastic gene expression. Nat Genet 37: 937–944. 16086016 10.1038/ng1616

[13] Elowitz MB, Levine AJ, Siggia ED, Swain PS (2002) Stochastic gene expression in a single cell. Science 297: 1183–1186. 12183631 10.1126/science.1070919

[14] McCullough MJ, Ross BC, Reade PC (1996) Candida albicans: a review of its history, taxonomy, epidemiology, virulence attributes, and methods of strain differentiation. Int J Oral Maxillofac Surg 25: 136–144. 8727588 10.1016/s0901-5027(96)80060-9

[15] Bezerra AR, Simoes J, Lee W, Rung J, Weil T, et al (2013) Reversion of a fungal genetic code alteration links proteome instability with genomic and phenotypic diversification. Proc Natl Acad Sci U S A 110: 11079–11084. 23776239 10.1073/pnas.1302094110 PMC3704024

[16] Selmecki AM, Dulmage K, Cowen LE, Anderson JB, Berman J (2009) Acquisition of aneuploidy provides increased fitness during the evolution of antifungal drug resistance. PLoS Genet 5: e1000705. 19876375 10.1371/journal.pgen.1000705 PMC2760147

[17] Arbour M, Epp E, Hogues H, Sellam A, Lacroix C, et al (2009) Widespread occurrence of chromosomal aneuploidy following the routine production of Candida albicans mutants. FEMS Yeast Res 9: 1070–1077. 19732157 10.1111/j.1567-1364.2009.00563.x PMC2784216

[18] Selmecki A, Forche A, Berman J (2006) Aneuploidy and isochromosome formation in drug-resistant Candida albicans. Science 313: 367–370. 16857942 10.1126/science.1128242 PMC1717021

[19] Yona AH, Manor YS, Herbst RH, Romano GH, Mitchell A, et al (2012) Chromosomal duplication is a transient evolutionary solution to stress. Proc Natl Acad Sci U S A 109: 21010–21015. 23197825 10.1073/pnas.1211150109 PMC3529009

[20] Sionov E, Chang YC, Kwon-Chung KJ (2013) Azole Heteroresistance in Cryptococcus neoformans: Emergence of Resistant Clones with Chromosomal Disomy in the Mouse Brain during Fluconazole Treatment. Antimicrob Agents Chemother 57 10: 5127–30. 23836187 10.1128/AAC.00694-13 PMC3811407

[21] Pavelka N, Rancati G, Zhu J, Bradford WD, Saraf A, et al (2010) Aneuploidy confers quantitative proteome changes and phenotypic variation in budding yeast. Nature 468: 321–325. 20962780 10.1038/nature09529 PMC2978756

[22] Selmecki A, Forche A, Berman J (2010) Genomic plasticity of the human fungal pathogen Candida albicans. Eukaryot Cell 9: 991–1008. 20495058 10.1128/EC.00060-10 PMC2901674

[23] Singh-Babak SD, Babak T, Diezmann S, Hill JA, Xie JL, et al (2012) Global analysis of the evolution and mechanism of echinocandin resistance in Candida glabrata. PLoS Pathog 8: e1002718. 22615574 10.1371/journal.ppat.1002718 PMC3355103

[24] Cowen LE, Anderson JB, Kohn LM (2002) Evolution of drug resistance in Candida albicans. Annu Rev Microbiol 56: 139–165. 12142485 10.1146/annurev.micro.56.012302.160907

[25] Magee BB, Hube B, Wright RJ, Sullivan PJ, Magee PT (1993) The genes encoding the secreted aspartyl proteinases of Candida albicans constitute a family with at least three members. Infect Immun 61: 3240–3243. 8335356 10.1128/iai.61.8.3240-3243.1993 PMC280994

[26] Hube B, Stehr F, Bossenz M, Mazur A, Kretschmar M, et al (2000) Secreted lipases of Candida albicans: cloning, characterisation and expression analysis of a new gene family with at least ten members. Arch Microbiol 174: 362–374. 11131027 10.1007/s002030000218

[27] Hoyer LL (2001) The ALS gene family of Candida albicans. Trends Microbiol 9: 176–180. 11286882 10.1016/s0966-842x(01)01984-9

[28] Jackson AP, Gamble JA, Yeomans T, Moran GP, Saunders D, et al (2009) Comparative genomics of the fungal pathogens Candida dubliniensis and Candida albicans. Genome Res 19: 2231–2244. 19745113 10.1101/gr.097501.109 PMC2792176

[29] Butler G, Rasmussen MD, Lin MF, Santos MA, Sakthikumar S, et al (2009) Evolution of pathogenicity and sexual reproduction in eight Candida genomes. Nature 459: 657–662. 19465905 10.1038/nature08064 PMC2834264

[30] van het Hoog M, Rast TJ, Martchenko M, Grindle S, Dignard D, et al (2007) Assembly of the Candida albicans genome into sixteen supercontigs aligned on the eight chromosomes. Genome Biol 8: R52. 17419877 10.1186/gb-2007-8-4-r52 PMC1896002

[31] Zhang A, Petrov KO, Hyun ER, Liu Z, Gerber SA, et al (2012) The Tlo proteins are stoichiometric components of Candida albicans mediator anchored via the Med3 subunit. Eukaryot Cell 11: 874–884. 22562472 10.1128/EC.00095-12 PMC3416505

[32] Mondoux MA, Zakian VA (2007) Subtelomeric elements influence but do not determine silencing levels at Saccharomyces cerevisiae telomeres. Genetics 177: 2541–2546. 18073447 10.1534/genetics.107.079806 PMC2219501

[33] Gottschling DE, Aparicio OM, Billington BL, Zakian VA (1990) Position effect at S. cerevisiae telomeres: reversible repression of Pol II transcription. Cell 63: 751–762. 2225075 10.1016/0092-8674(90)90141-z

[34] Blackburn EH (2001) Switching and signaling at the telomere. Cell 106: 661–673. 11572773 10.1016/s0092-8674(01)00492-5

[35] Oppikofer M, Kueng S, Gasser SM (2013) SIR-nucleosome interactions: Structure-function relationships in yeast silent chromatin. Gene 527 1: 10–25. 23791651 10.1016/j.gene.2013.05.088

[36] Doheny JG, Mottus R, Grigliatti TA (2008) Telomeric position effect–a third silencing mechanism in eukaryotes. PLoS One 3: e3864. 19057646 10.1371/journal.pone.0003864 PMC2587703

[37] Greiss S, Gartner A (2009) Sirtuin/Sir2 phylogeny, evolutionary considerations and structural conservation. Mol Cells 28: 407–415. 19936627 10.1007/s10059-009-0169-x PMC3710699

[38] Belenky P, Racette FG, Bogan KL, McClure JM, Smith JS, et al (2007) Nicotinamide riboside promotes Sir2 silencing and extends lifespan via Nrk and Urh1/Pnp1/Meu1 pathways to NAD+. Cell 129: 473–484. 17482543 10.1016/j.cell.2007.03.024

[39] Choi JK, Hwang S, Kim YJ (2008) Stochastic and regulatory role of chromatin silencing in genomic response to environmental changes. PLoS One 3: e3002. 18714342 10.1371/journal.pone.0003002 PMC2500160

[40] Weinberger L, Voichek Y, Tirosh I, Hornung G, Amit I, et al (2012) Expression noise and acetylation profiles distinguish HDAC functions. Mol Cell 47: 193–202. 22683268 10.1016/j.molcel.2012.05.008 PMC3408861

[41] Tirosh I, Weinberger A, Carmi M, Barkai N (2006) A genetic signature of interspecies variations in gene expression. Nat Genet 38: 830–834. 16783381 10.1038/ng1819

[42] Chen M, Licon K, Otsuka R, Pillus L, Ideker T (2013) Decoupling epigenetic and genetic effects through systematic analysis of gene position. Cell Rep 3: 128–137. 23291096 10.1016/j.celrep.2012.12.003 PMC3563736

[43] Perez-Martin J, Uria JA, Johnson AD (1999) Phenotypic switching in Candida albicans is controlled by a SIR2 gene. EMBO J 18: 2580–2592. 10228170 10.1093/emboj/18.9.2580 PMC1171338

[44] Stevenson JS, Liu H (2011) Regulation of white and opaque cell-type formation in Candida albicans by Rtt109 and Hst3. Mol Microbiol 81: 1078–1091. 21749487 10.1111/j.1365-2958.2011.07754.x PMC4049571

[45] Hnisz D, Schwarzmuller T, Kuchler K (2009) Transcriptional loops meet chromatin: a dual-layer network controls white-opaque switching in Candida albicans. Mol Microbiol 74: 1–15. 19555456 10.1111/j.1365-2958.2009.06772.x PMC2764112

[46] Hnisz D, Bardet AF, Nobile CJ, Petryshyn A, Glaser W, et al (2012) A histone deacetylase adjusts transcription kinetics at coding sequences during Candida albicans morphogenesis. PLoS Genet 8: e1003118. 23236295 10.1371/journal.pgen.1003118 PMC3516536

[47] Cormack BP, Ghori N, Falkow S (1999) An adhesin of the yeast pathogen Candida glabrata mediating adherence to human epithelial cells. Science 285: 578–582. 10417386 10.1126/science.285.5427.578

[48] Halliwell SC, Smith MC, Muston P, Holland SL, Avery SV (2012) Heterogeneous expression of the virulence-related adhesin Epa1 between individual cells and strains of the pathogen Candida glabrata. Eukaryot Cell 11: 141–150. 22140233 10.1128/EC.05232-11 PMC3272907

[49] Rosin D, Hornung G, Tirosh I, Gispan A, Barkai N (2012) Promoter nucleosome organization shapes the evolution of gene expression. PLoS Genet 8: e1002579. 22438828 10.1371/journal.pgen.1002579 PMC3305400

[50] Bruno VM, Wang Z, Marjani SL, Euskirchen GM, Martin J, et al (2010) Comprehensive annotation of the transcriptome of the human fungal pathogen Candida albicans using RNA-seq. Genome Res 20: 1451–1458. 20810668 10.1101/gr.109553.110 PMC2945194

[51] Anderson MZ, Baller JA, Dulmage K, Wigen L, Berman J (2012) The three clades of the telomere-associated TLO gene family of Candida albicans have different splicing, localization, and expression features. Eukaryot Cell 11: 1268–1275. 22923044 10.1128/EC.00230-12 PMC3485924

[52] Piccirillo S, White MG, Murphy JC, Law DJ, Honigberg SM (2010) The Rim101p/PacC pathway and alkaline pH regulate pattern formation in yeast colonies. Genetics 184: 707–716. 20038633 10.1534/genetics.109.113480 PMC2845339

[53] Purnapatre K, Honigberg SM (2002) Meiotic differentiation during colony maturation in Saccharomyces cerevisiae. Curr Genet 42: 1–8. 12420140 10.1007/s00294-002-0331-x

[54] Field Y, Kaplan N, Fondufe-Mittendorf Y, Moore IK, Sharon E, et al (2008) Distinct modes of regulation by chromatin encoded through nucleosome positioning signals. PLoS Comput Biol 4: e1000216. 18989395 10.1371/journal.pcbi.1000216 PMC2570626

[55] Zaugg JB, Luscombe NM (2012) A genomic model of condition-specific nucleosome behavior explains transcriptional activity in yeast. Genome Res 22: 84–94. 21930892 10.1101/gr.124099.111 PMC3246209

[56] Tan RZ, van Oudenaarden A (2010) Transcript counting in single cells reveals dynamics of rDNA transcription. Mol Syst Biol 6: 358. 20393578 10.1038/msb.2010.14 PMC2872610

[57] Kaeberlein M, McVey M, Guarente L (1999) The SIR2/3/4 complex and SIR2 alone promote longevity in Saccharomyces cerevisiae by two different mechanisms. Genes Dev 13: 2570–2580. 10521401 10.1101/gad.13.19.2570 PMC317077

[58] Halme A, Bumgarner S, Styles C, Fink GR (2004) Genetic and epigenetic regulation of the FLO gene family generates cell-surface variation in yeast. Cell 116: 405–415. 15016375 10.1016/s0092-8674(04)00118-7

[59] Li X, Zhao X, Fang Y, Jiang X, Duong T, et al (1998) Generation of destabilized green fluorescent protein as a transcription reporter. J Biol Chem 273: 34970–34975. 9857028 10.1074/jbc.273.52.34970

[60] Kaiser B, Munder T, Saluz HP, Kunkel W, Eck R (1999) Identification of a gene encoding the pyruvate decarboxylase gene regulator CaPdc2p from Candida albicans. Yeast 15: 585–591. 10341421 10.1002/(SICI)1097-0061(199905)15:7<585::AID-YEA401>3.0.CO;2-9

[61] Inigo S, Giraldez AN, Chory J, Cerdan PD (2012) Proteasome-mediated turnover of Arabidopsis MED25 is coupled to the activation of FLOWERING LOCUS T transcription. Plant Physiol 160: 1662–1673. 22992513 10.1104/pp.112.205500 PMC3490578

[62] Molinari E, Gilman M, Natesan S (1999) Proteasome-mediated degradation of transcriptional activators correlates with activation domain potency in vivo. EMBO J 18: 6439–6447. 10562555 10.1093/emboj/18.22.6439 PMC1171706

[63] Verstrepen KJ, Fink GR (2009) Genetic and epigenetic mechanisms underlying cell-surface variability in protozoa and fungi. Annu Rev Genet 43: 1–24. 19640229 10.1146/annurev-genet-102108-134156

[64] Lehner B (2010) Conflict between noise and plasticity in yeast. PLoS Genet 6: e1001185. 21079670 10.1371/journal.pgen.1001185 PMC2973811

[65] Carey LB, van Dijk D, Sloot PM, Kaandorp JA, Segal E (2013) Promoter sequence determines the relationship between expression level and noise. PLoS Biol 11: e1001528. 23565060 10.1371/journal.pbio.1001528 PMC3614515

[66] Freeman-Cook LL, Sherman JM, Brachmann CB, Allshire RC, Boeke JD, et al (1999) The Schizosaccharomyces pombe hst4(+) gene is a SIR2 homologue with silencing and centromeric functions. Mol Biol Cell 10: 3171–3186. 10512858 10.1091/mbc.10.10.3171 PMC25575

[67] Freitas-Junior LH, Hernandez-Rivas R, Ralph SA, Montiel-Condado D, Ruvalcaba-Salazar OK, et al (2005) Telomeric heterochromatin propagation and histone acetylation control mutually exclusive expression of antigenic variation genes in malaria parasites. Cell 121: 25–36. 15820676 10.1016/j.cell.2005.01.037

[68] Pirrotta V, Gross DS (2005) Epigenetic silencing mechanisms in budding yeast and fruit fly: different paths, same destinations. Mol Cell 18: 395–398. 15893722 10.1016/j.molcel.2005.04.013

[69] Kitada T, Kuryan BG, Tran NN, Song C, Xue Y, et al (2012) Mechanism for epigenetic variegation of gene expression at yeast telomeric heterochromatin. Genes Dev 26: 2443–2455. 23124068 10.1101/gad.201095.112 PMC3490002

[70] Rossmann MP, Luo W, Tsaponina O, Chabes A, Stillman B (2011) A common telomeric gene silencing assay is affected by nucleotide metabolism. Mol Cell 42: 127–136. 21474074 10.1016/j.molcel.2011.03.007 PMC3086572

[71] Zhu X, Zhang Y, Bjornsdottir G, Liu Z, Quan A, et al (2011) Histone modifications influence mediator interactions with chromatin. Nucleic Acids Res 39: 8342–8354. 21742760 10.1093/nar/gkr551 PMC3201872

[72] Peng J, Zhou JQ (2012) The tail-module of yeast Mediator complex is required for telomere heterochromatin maintenance. Nucleic Acids Res 40: 581–593. 21930512 10.1093/nar/gkr757 PMC3258146

[73] Burrack LS, Applen SE, Berman J (2011) The requirement for the Dam1 complex is dependent upon the number of kinetochore proteins and microtubules. Curr Biol 21: 889–896. 21549601 10.1016/j.cub.2011.04.002 PMC3100407

[74] Gerami-Nejad M, Berman J, Gale CA (2001) Cassettes for PCR-mediated construction of green, yellow, and cyan fluorescent protein fusions in Candida albicans. Yeast 18: 859–864. 11427968 10.1002/yea.738

[75] Gerami-Nejad M, Forche A, McClellan M, Berman J (2012) Analysis of protein function in clinical C. albicans isolates. Yeast 29: 303–309. 22777821 10.1002/yea.2910 PMC3449217

[76] Greenbaum D, Colangelo C, Williams K, Gerstein M (2003) Comparing protein abundance and mRNA expression levels on a genomic scale. Genome Biol 4: 117. 12952525 10.1186/gb-2003-4-9-117 PMC193646

[77] R Development Core Team (2010) R: A language and environment for statistical computing. Vienna, Austria: R Foundation for Statistical Computing.

[78] Sudbery PE (2001) The germ tubes of Candida albicans hyphae and pseudohyphae show different patterns of septin ring localization. Mol Microbiol 41: 19–31. 11454197 10.1046/j.1365-2958.2001.02459.x

