# Supplementary figures and images for: Silencing Is Noisy: Population and Cell Level Noise in Telomere-Adjacent Genes Is Dependent on Telomere Position and Sir2
Source: PLoS Genet. 2014 Jul 24;10(7):e1004436. doi: 10.1371/journal.pgen.1004436 (PMC4109849; doi:10.1371/journal.pgen.1004436)

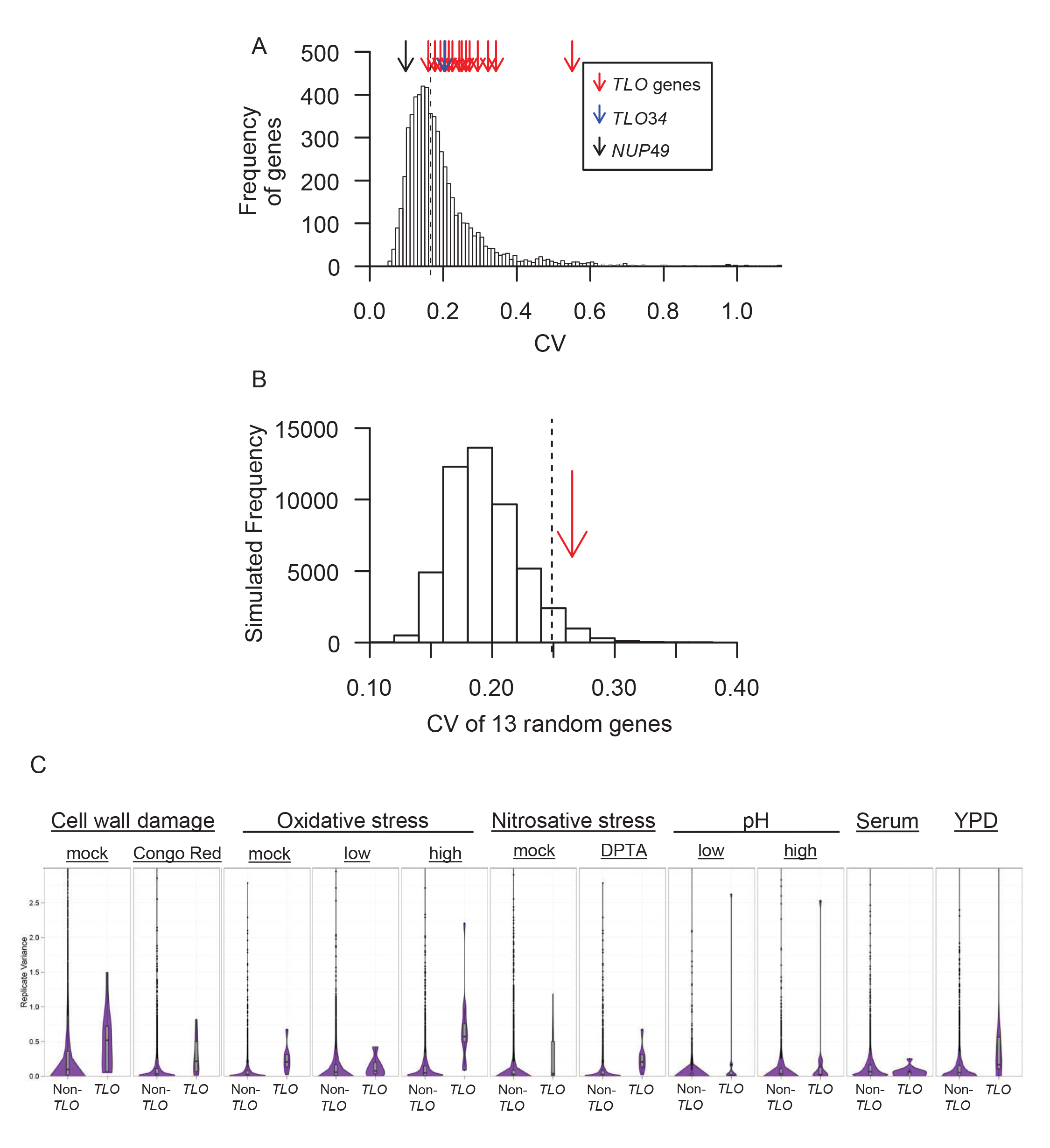

Supplement: Figure S1 — Elevated TLO transcriptional variation identified by RNA-seq. (A) The CV of all genes assayed by RNA-seq in Bruno, et al, was calculated averaging across all eleven conditions. The CV of the thirteen expressed TLOs is indicated by red arrows. (B) The average CV of TLO genes (red arrow) was compared against the CV from 50000 simulated datasets of 13 random genes. The 95% quantile of these datasets is indicated with the vertical dashed line. (C) The CV of each TLO (“TLO”) and all genes expressed within two standard deviations of TLO genes by RNA-seq (“non-TLO”) was plotted for each condition tested in Bruno, et al. (TIF) [file pgen.1004436.s001.tif]

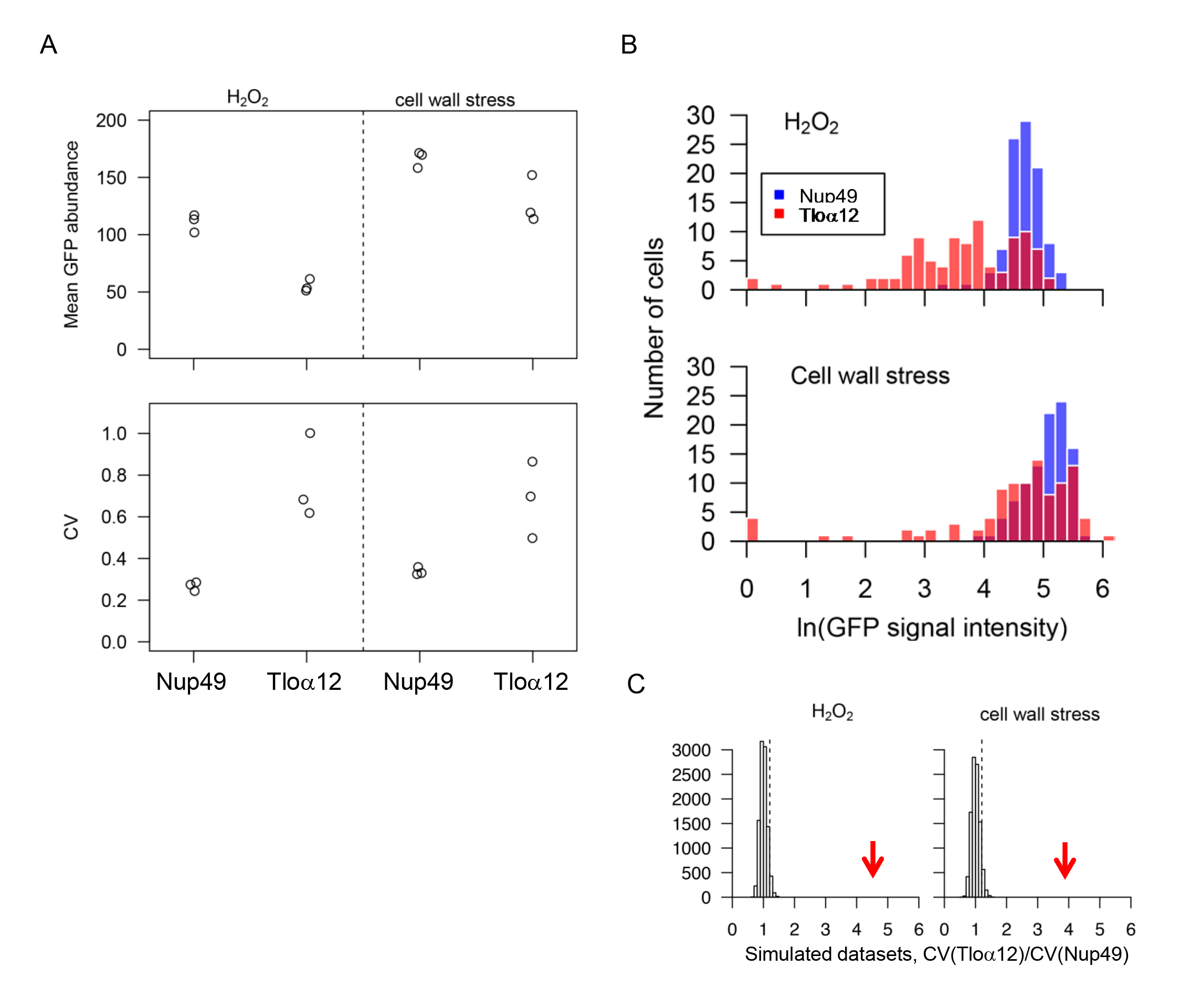

Supplement: Figure S2 — TLOs exhibit cell-to-cell variance under stress conditions. (A) GFP expression was quantified by microscopy for 150 cells from 3 biological replicates each of Nup49-GFP and Tloα12-GFP strains. The mean expression and CV were plotted for each replicate. (B) GFP expression of individual cells from (A) was quantified. (C) The ratio of Tloα12-GFP to Nup49-GFP CV was tested against simulated datasets constructed from all expression data for a single condition. GFP abundance was significantly more variable for Tloα12-GFP compared to Nup49-GFP. (TIF) [file pgen.1004436.s002.tif]

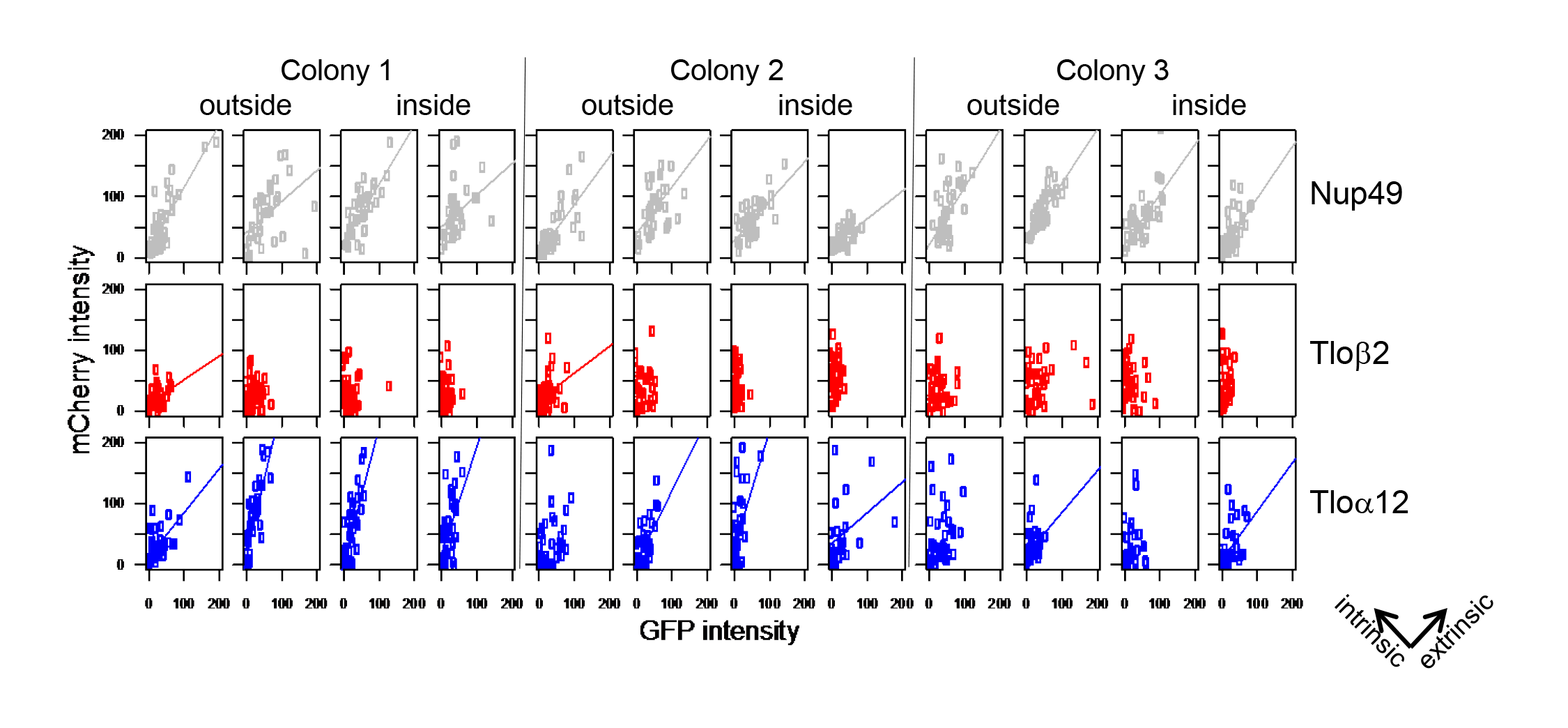

Supplement: Figure S4 — Subtelomeric TLOs have elevated intrinsic noise. Abundance of GFP and mCherry signal for single cells in Nup49, Tloβ2, and Tloα12-tagged cells was plotted for at least 50 cells from twelve biological replicates, four regions of three separate colonies. A best fit line and the intrinsic and extrinsic components of noise were calculated for each sample. Intrinsic noise was significantly greater for Tloα12 and TLOβ2 than for Nup49. (TIF) [file pgen.1004436.s004.tif]

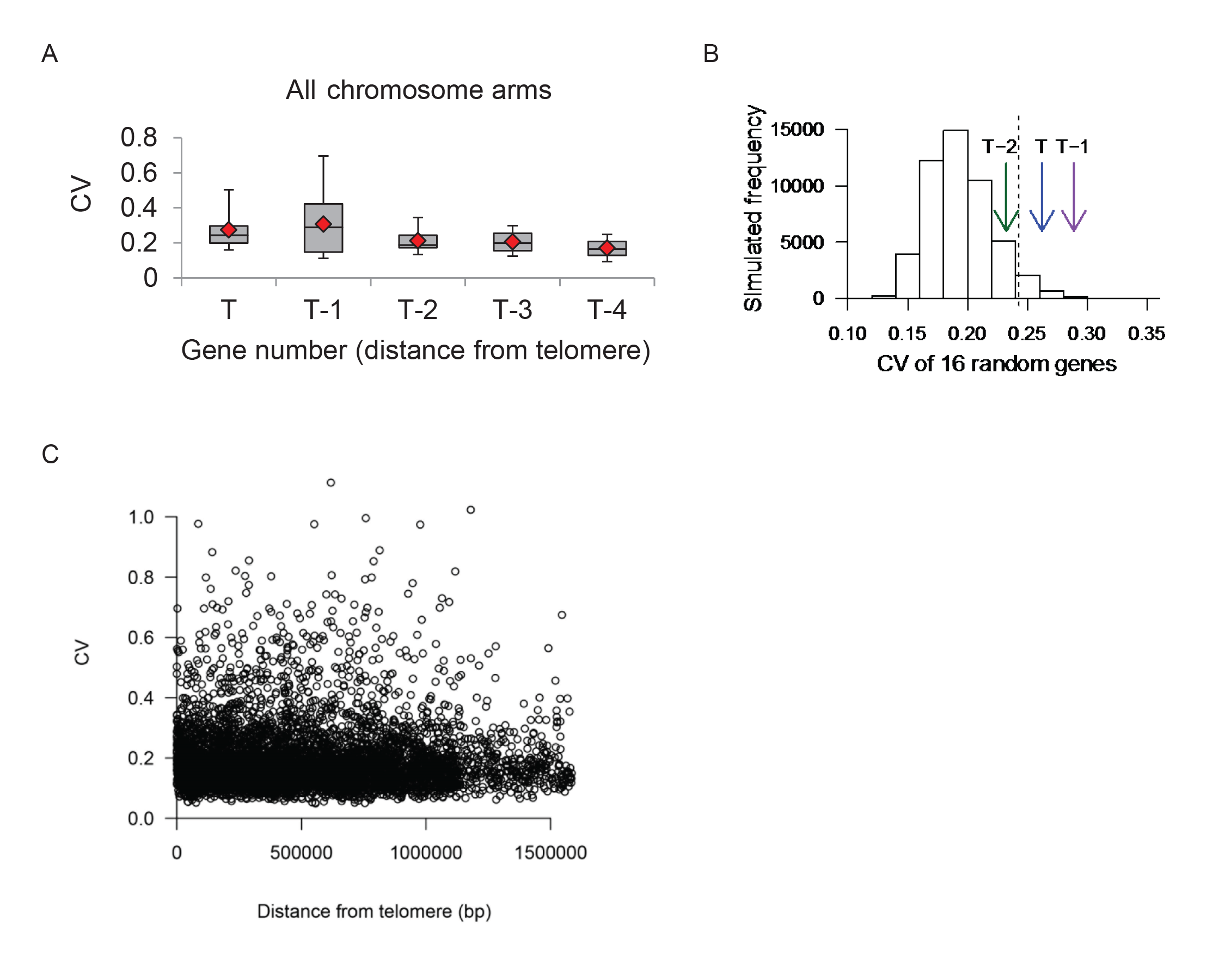

Supplement: Figure S5 — Expression plasticity is associated with telomere proximity. (A) The CV between replicates for the five most telomeric genes from each chromosome arm was averaged across all eleven condition tested in Bruno, et al. The CV of each telomere adjacent gene was compiled based on position and plotted from the most telomeric gene “T” to the most centromeric gene “T-5”. The red diamond indicates the mean CV for that position. (B) The average CV of the 16 most telomeric genes (“T”, blue arrow), 16 telomeric-1 genes (“T-1”, purple arrow), and 16 telomeric+2 genes (“T-2”, green arrow) was compared against the CV from 50000 simulated datasets of 16 random genes. (C) The average CV for all genes was plotted against their genomic position. A small but significant decrease in noise was identified with increased distance from the centromere. (TIF) [file pgen.1004436.s005.tif]

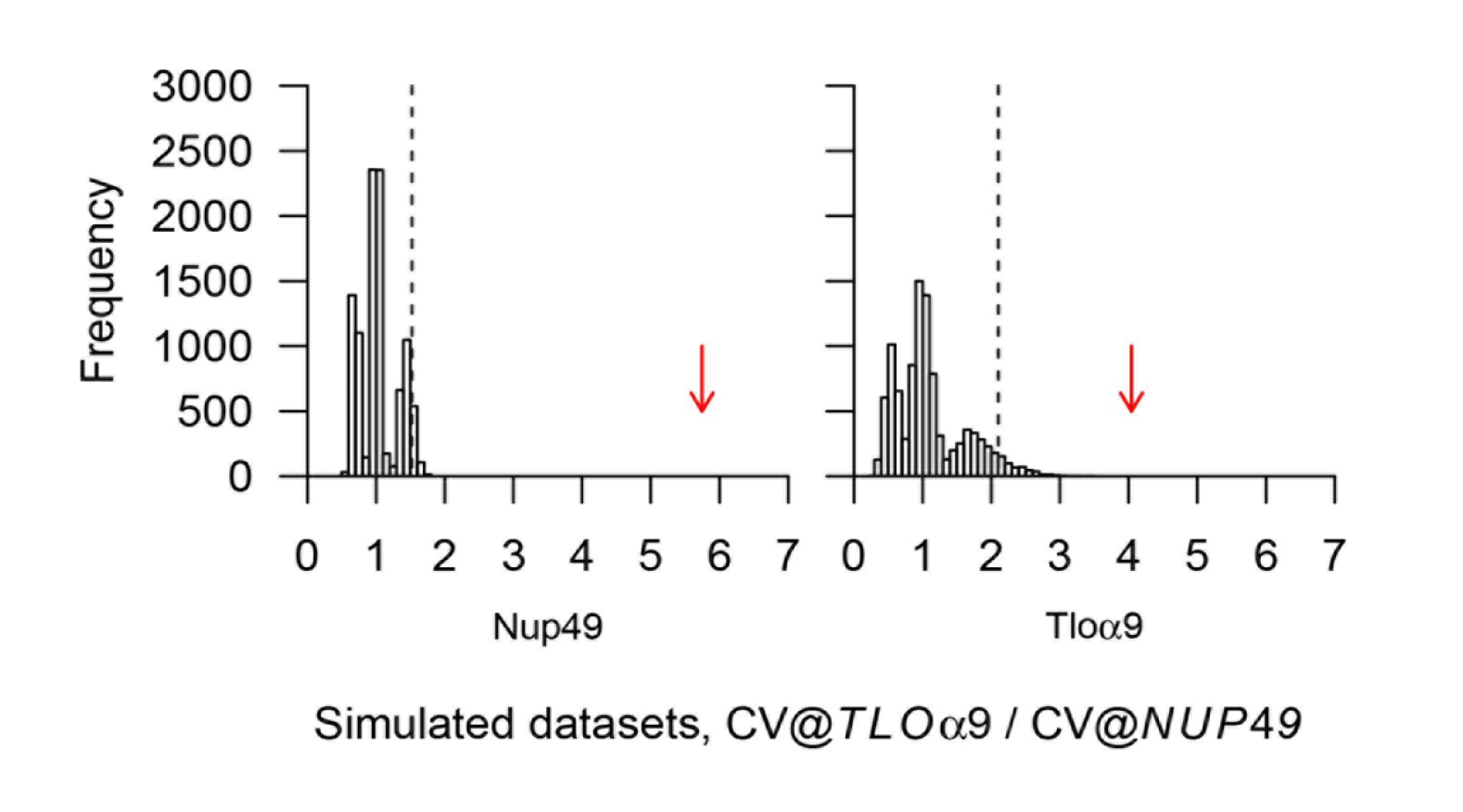

Supplement: Figure S6 — Gene noise increases at the subtelomere. Significance of the coefficient of variation ratio between expression of either gene at the TLOα9 and NUP49 locus was tested compared to randomized assignment of gene expression at the two loci. The noise ratio of the collected expression data was beyond the critical value (dashed line) indicating significantly elevated noise at the subtelomeric TLOα9 locus. (TIF) [file pgen.1004436.s006.tif]

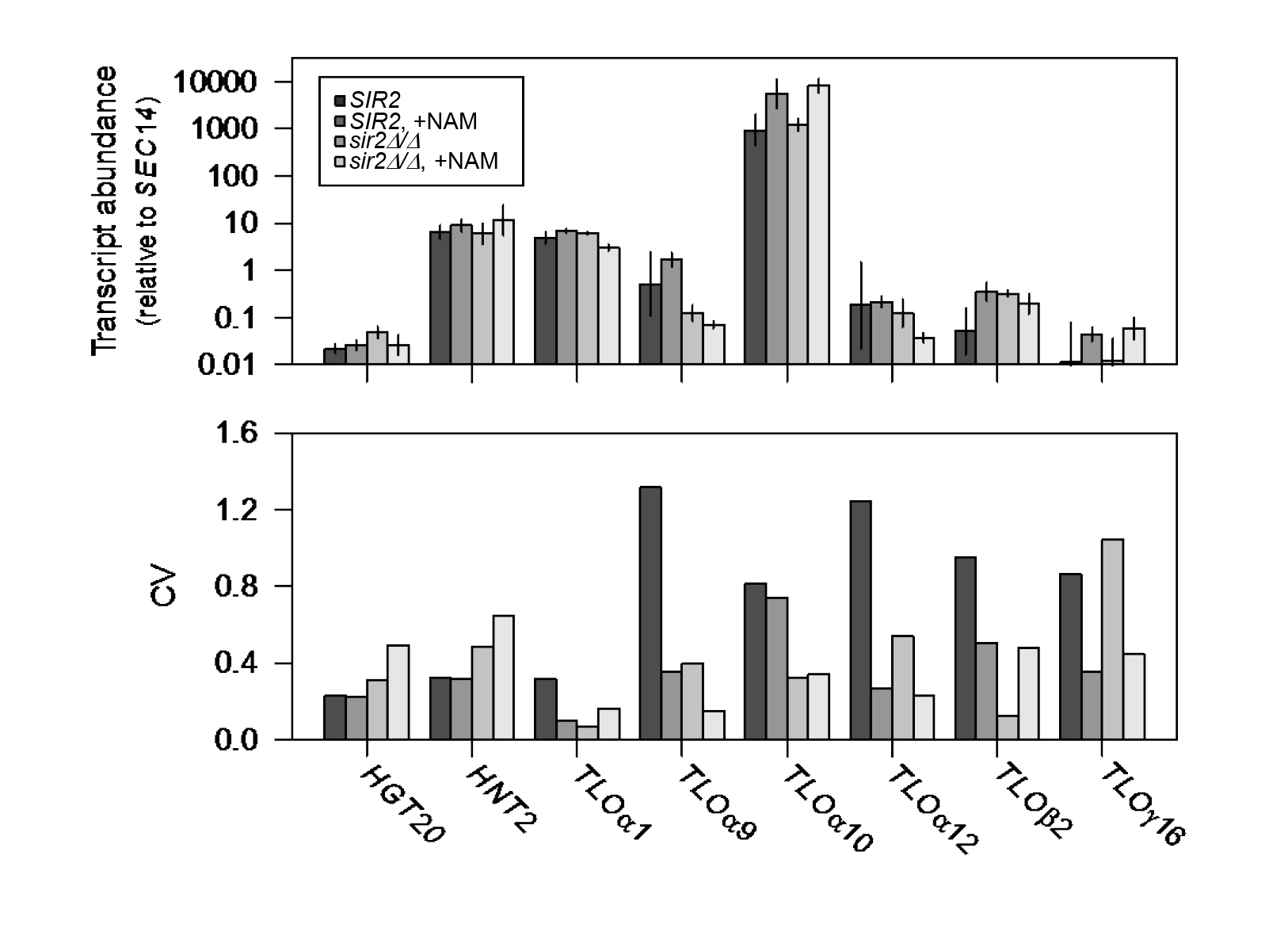

Supplement: Figure S7 — Gene expression plasticity of individual TLOs is affected by Sir-type HDAC function. The transcript abundance and CV of two control genes, SOD2 and HGT20, and six subtelomeric TLOs was plotted from either SIR2 or sir2Δ/Δ cells and in the presence or absence of nicotinamide. (TIF) [file pgen.1004436.s007.tif]

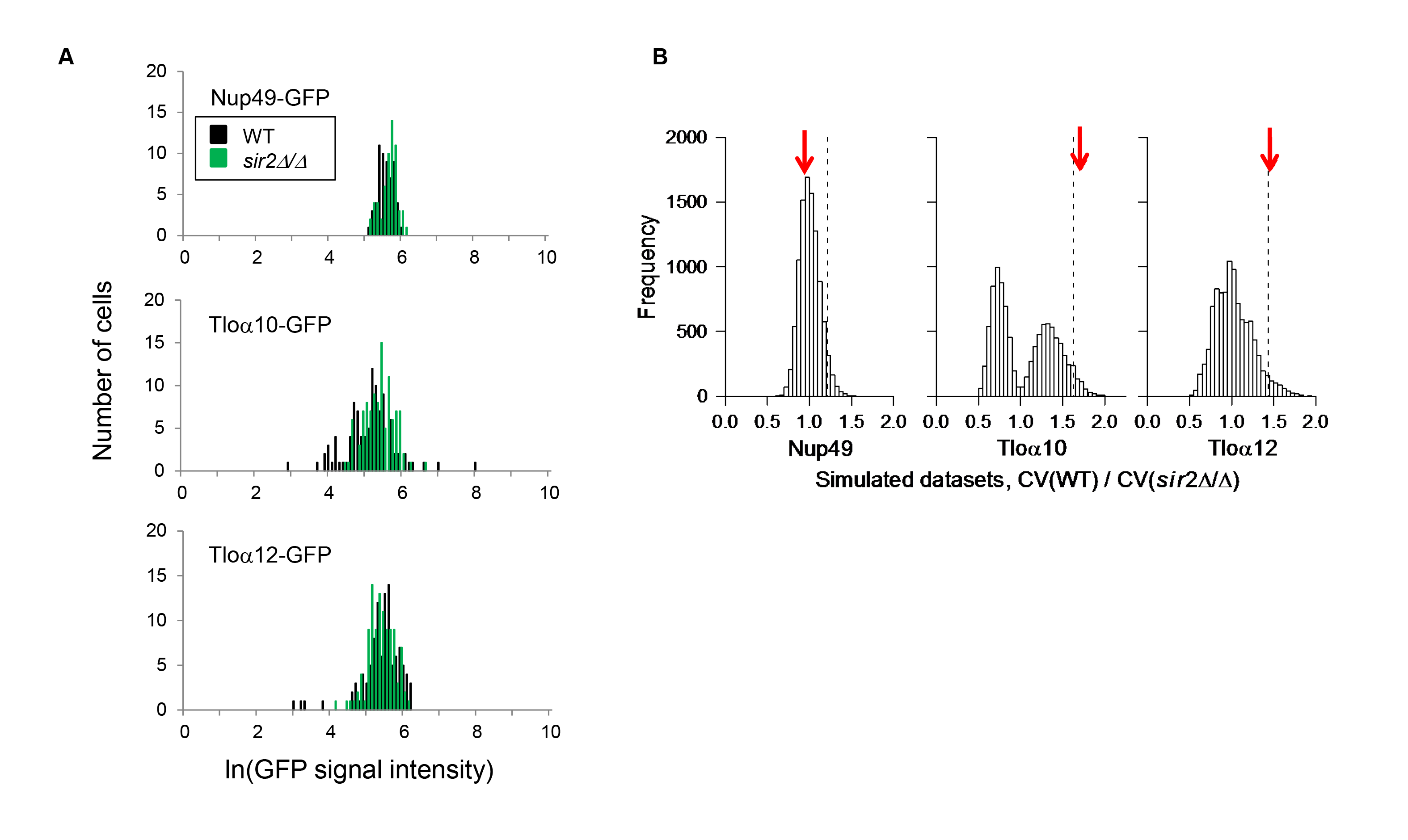

Supplement: Figure S8 — SIR2 contributes to Tlo noise. GFP expression was quantified by microscopy as shown in Figure 6B for 78 cells from 2 biological replicates (A) and the ratio of the CV in the WT to the sir2Δ/Δ background was tested against simulated datasets (B) constructed from all expression data for a single gene in either background. Analysis of the expression data identified significantly reduced noise for Tloα10 and Tloα12 associated with deletion of SIR2 but not for Nup49. (TIF) [file pgen.1004436.s008.tif]

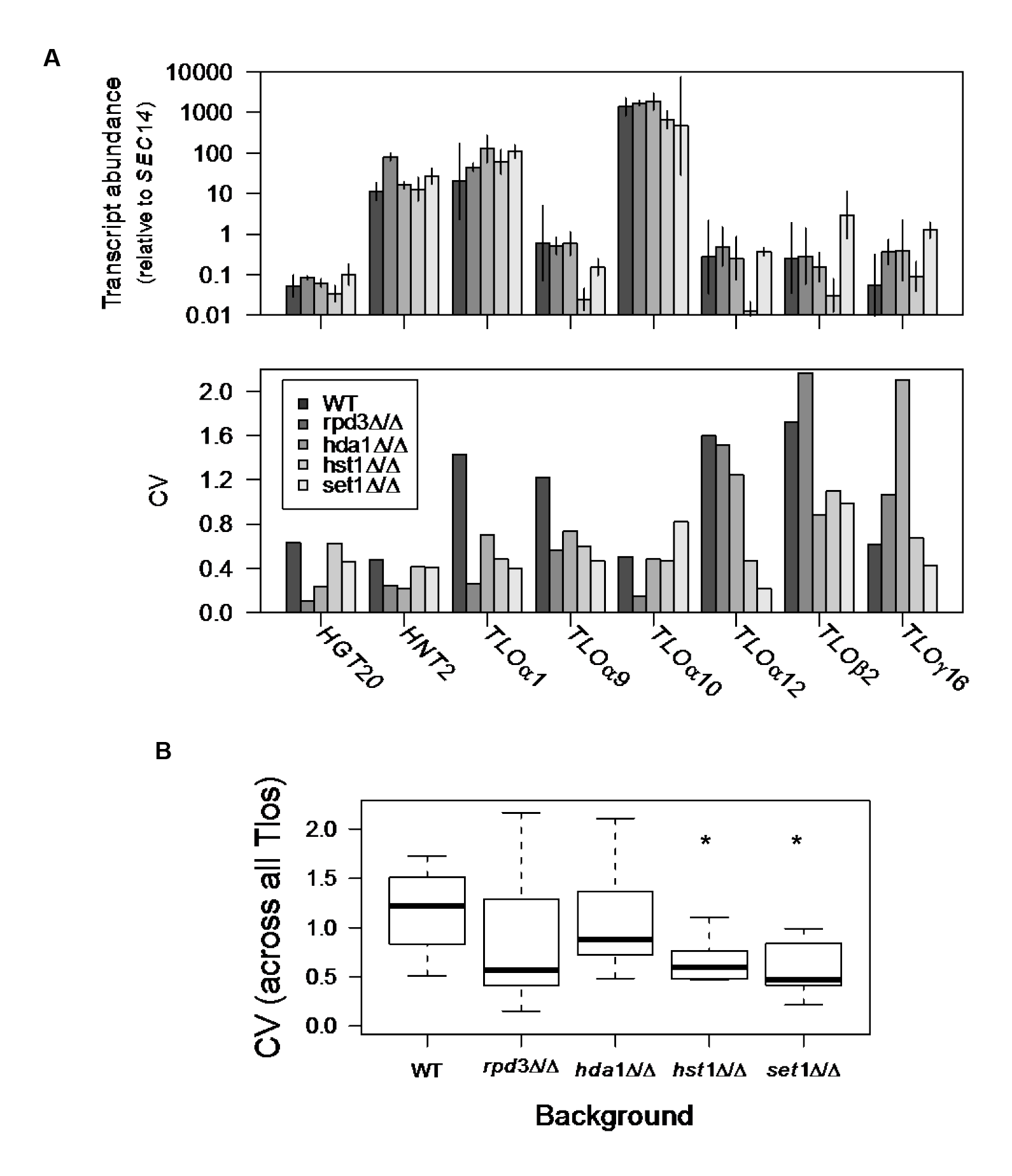

Supplement: Figure S9 — Expression plasticity of individual TLOs is affected by additional chromatin modifiers. The (A) transcript abundance and CV of two control genes and six subtelomeric TLOs was plotted from either WT, rpd3Δ/Δ, hda1Δ/Δ, hst1Δ/Δ, or set1Δ/Δ cells. (B)Expression variability of TLOs was significantly reduced by deletion of HST1 and SET1. (TIF) [file pgen.1004436.s009.tif]

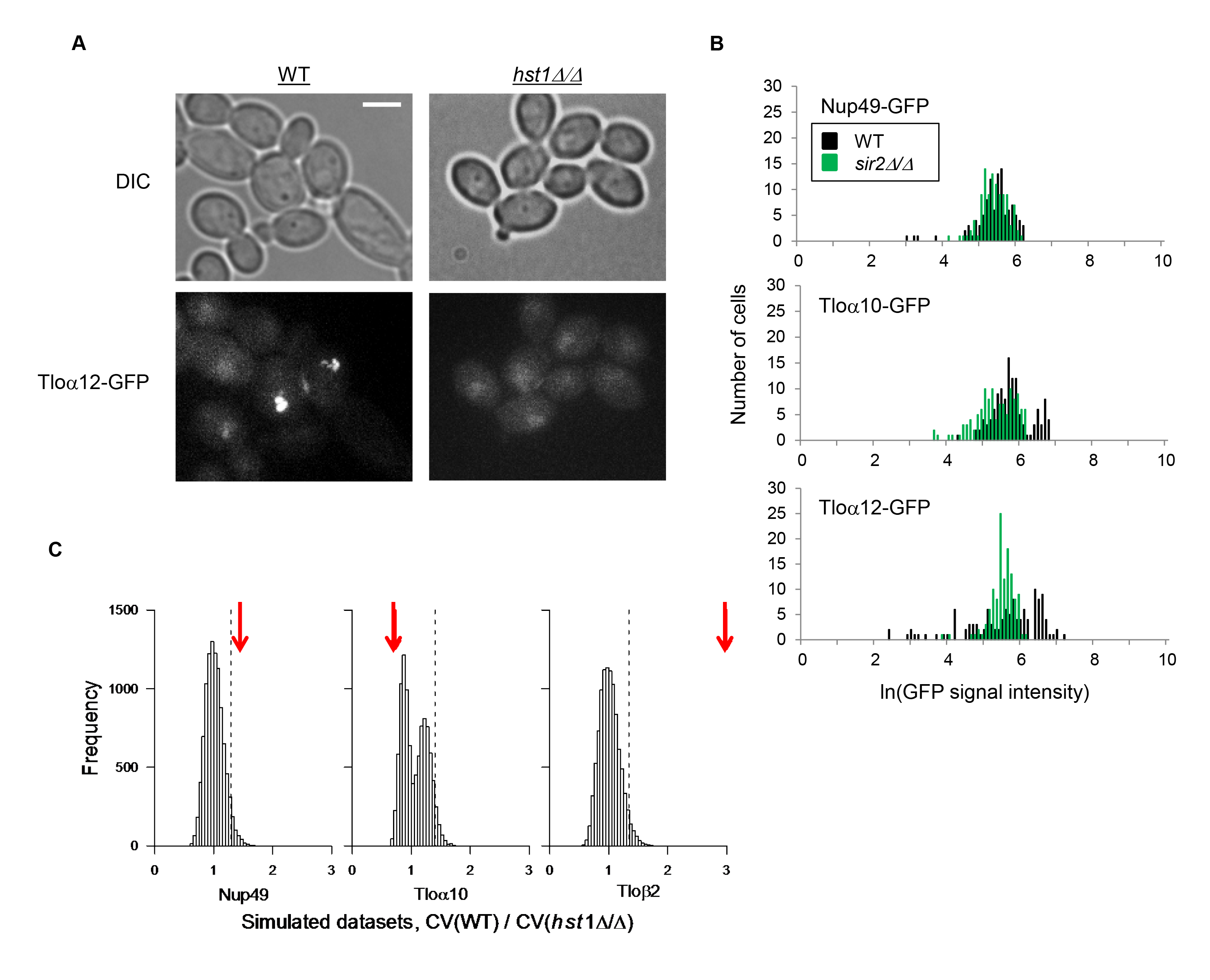

Supplement: Figure S10 — Hst1 and Set1 influence gene noise. (A–C) Fluorescence microscopy (A) analysis of GFP-tagged Tlos and Nup49 was performed in either a HST1 or hst1Δ/Δ background. GFP expression was quantified (B) for 100 cells from 2 biological replicates and the ratio of the CV in the WT to the hst1Δ/Δ background was tested against simulated datasets (C) constructed from all expression data for a single gene in either background. Analysis of the expression data identified significantly reduced fluorescence signal for both Tlos and reduced noise for Tloα12 but not Tloα10 in the hst1Δ/Δ background. Noise was also reduced for Nup49 in the hst1Δ/Δ background. (TIF) [file pgen.1004436.s010.tif]

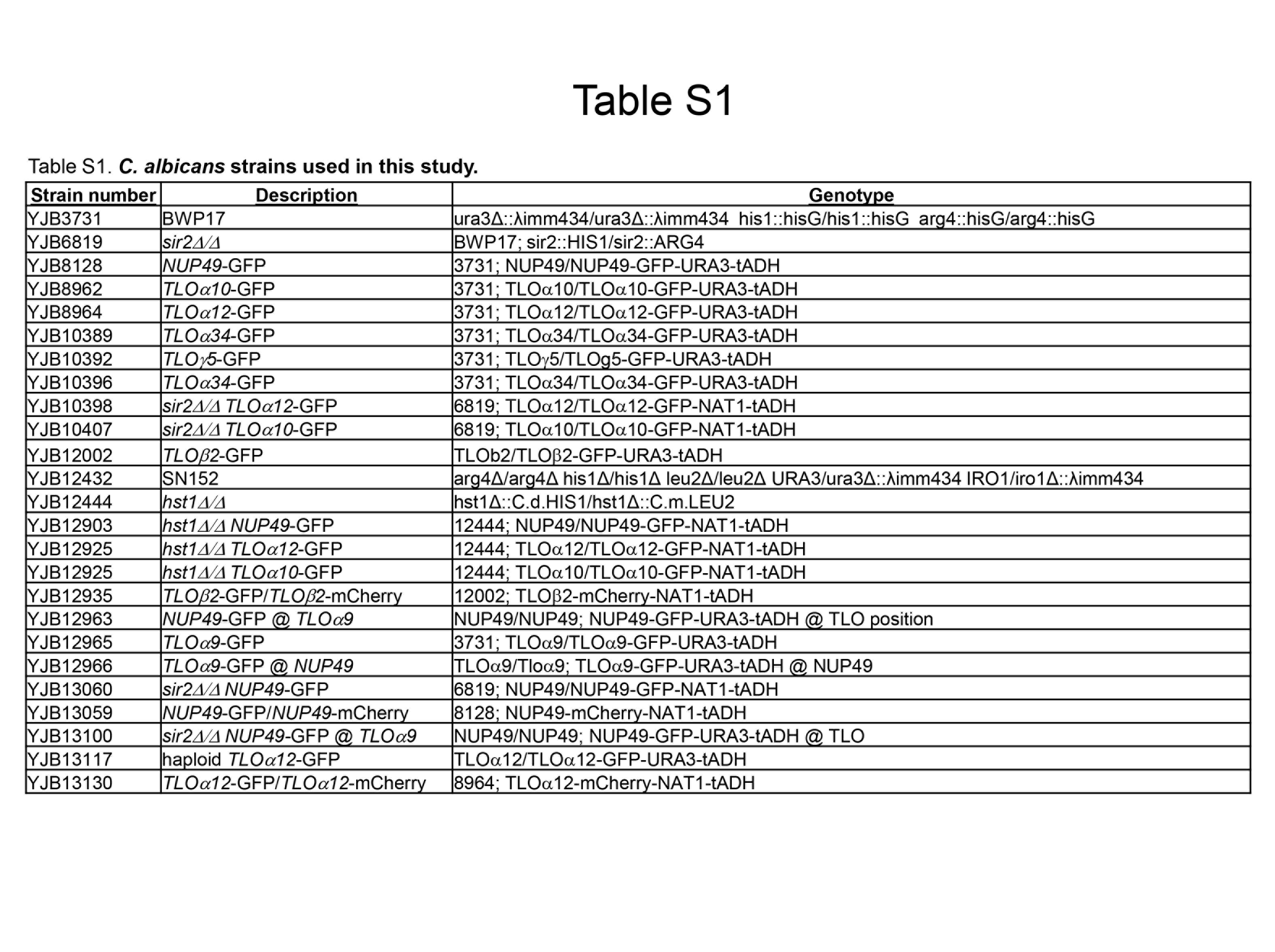

Supplement: Table S1 — C. albicans strains used in this study. (TIF) [file pgen.1004436.s012.tif]

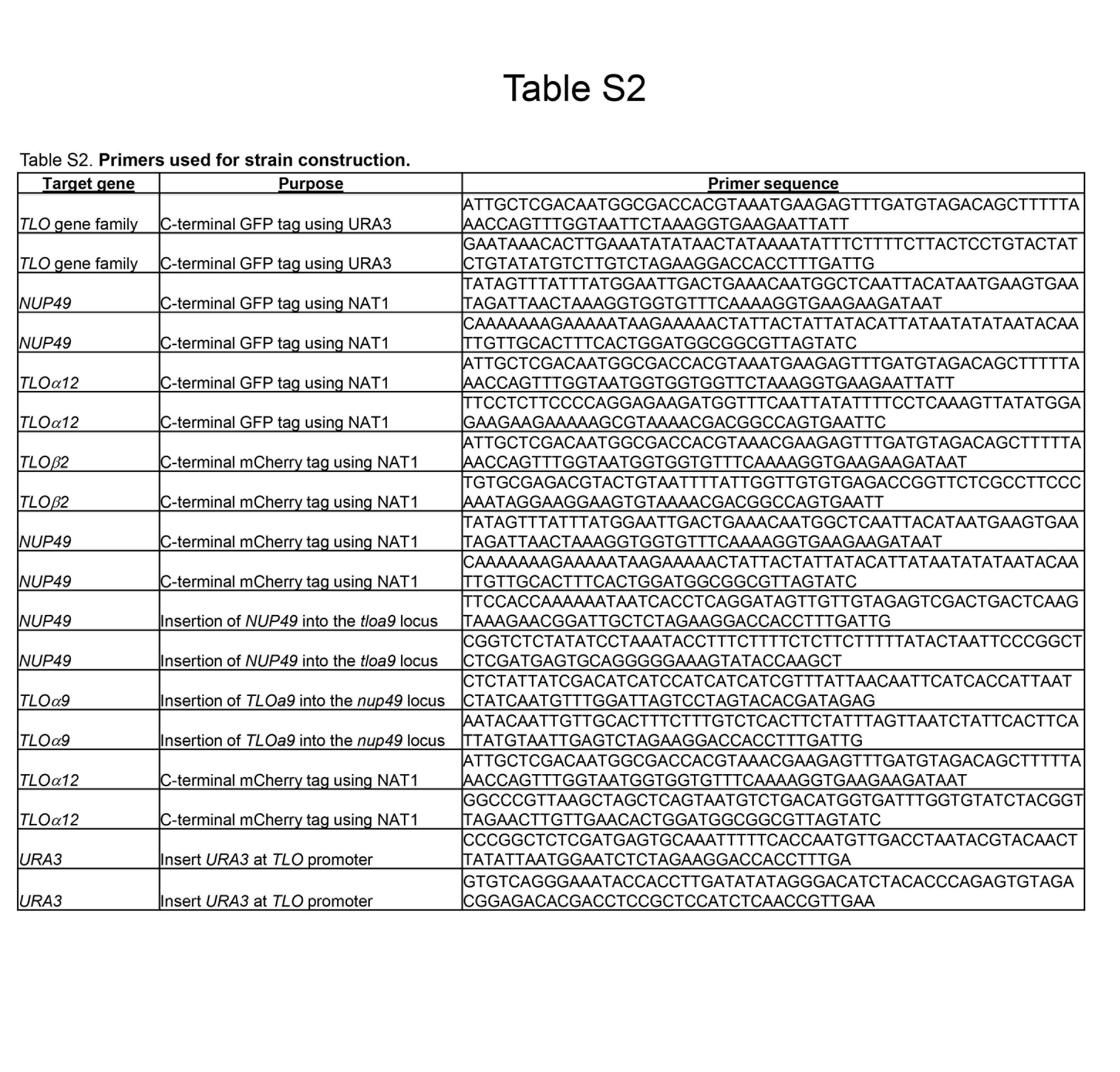

Supplement: Table S2 — Primers used for strain construction. (TIF) [file pgen.1004436.s013.tif]

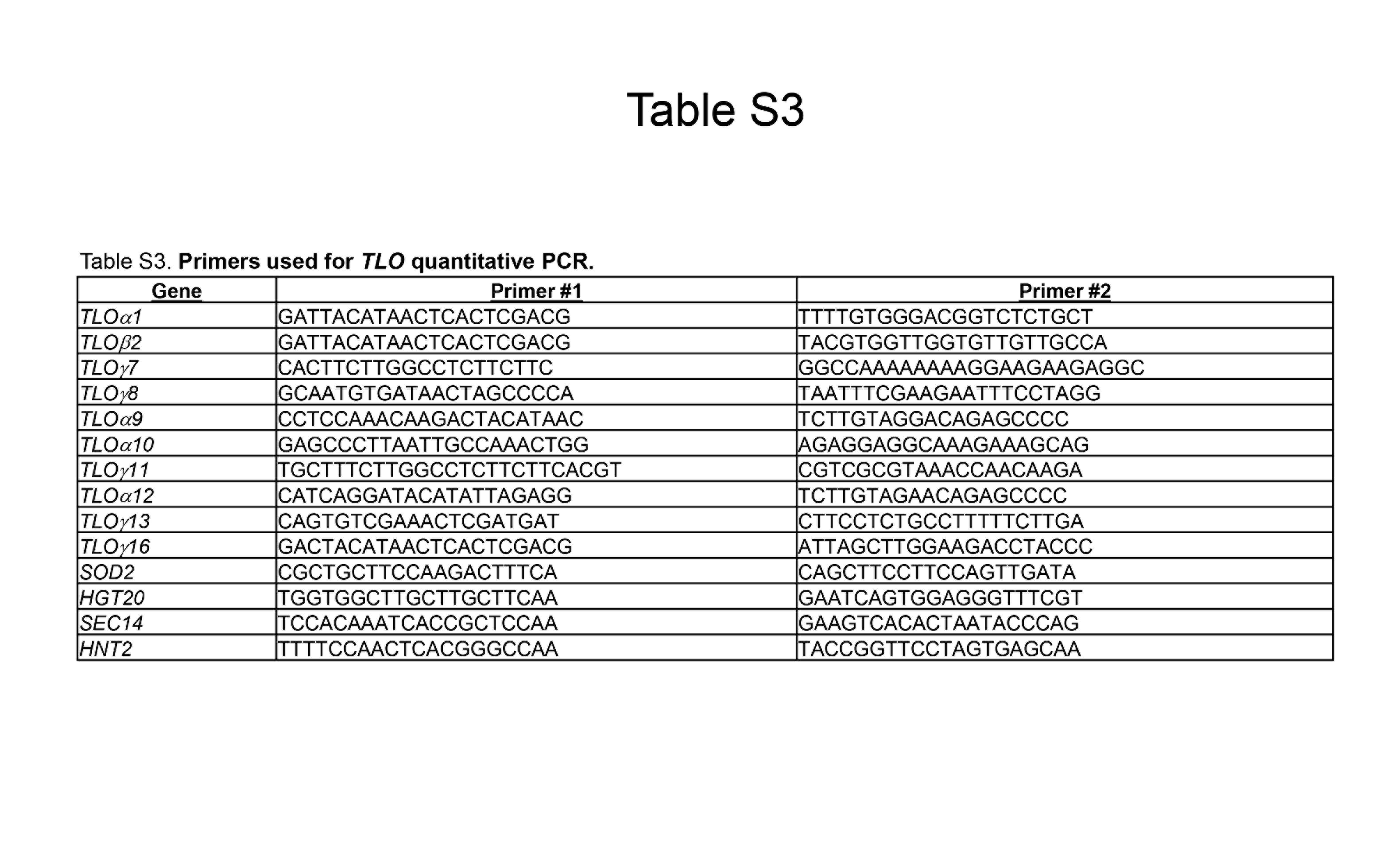

Supplement: Table S3 — Primers used for TLO quantitative PCR. (TIF) [file pgen.1004436.s014.tif]

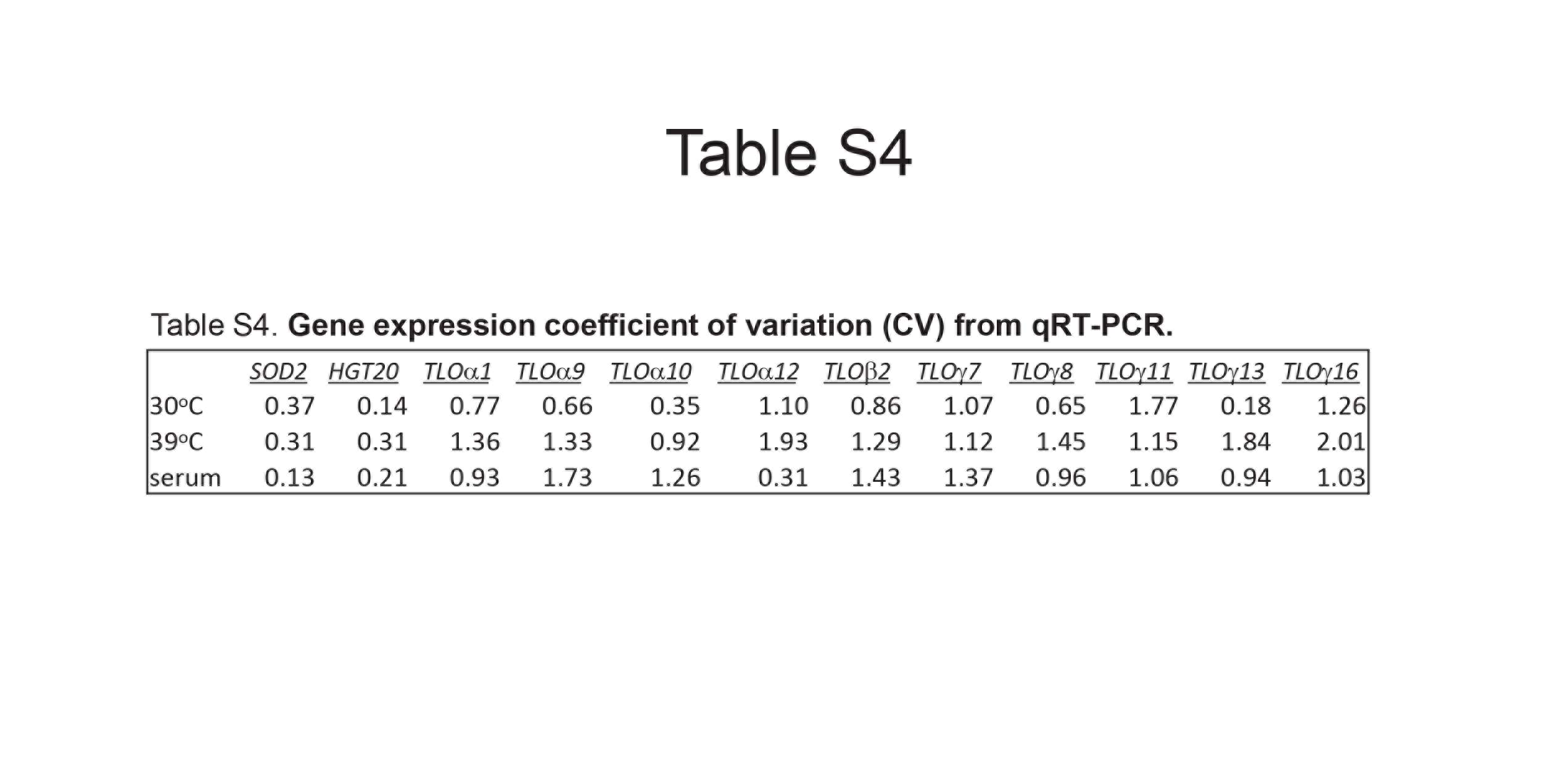

Supplement: Table S4 — Gene expression coefficient of variation (CV) from qRT-PCR. (TIF) [file pgen.1004436.s015.tif]

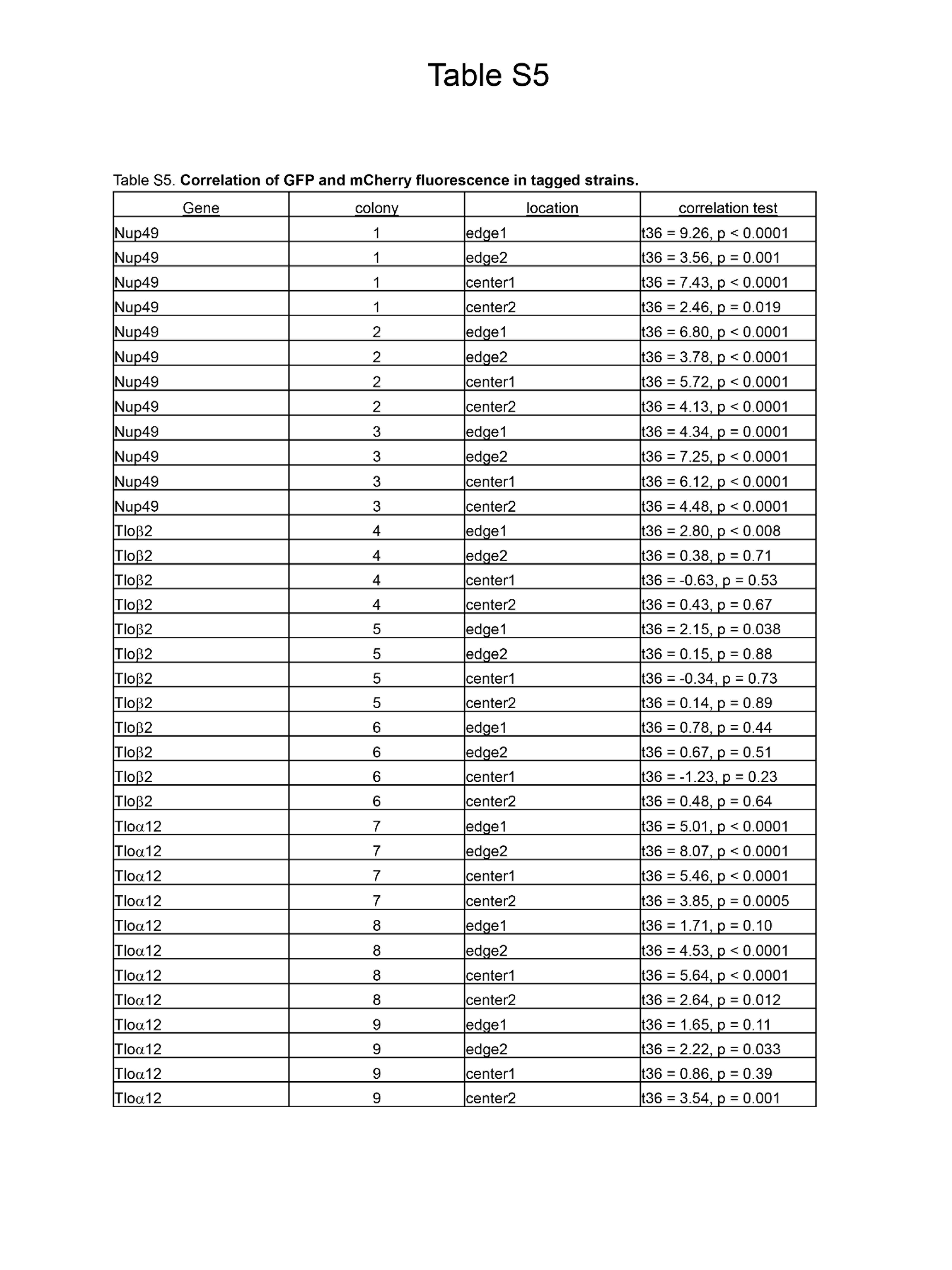

Supplement: Table S5 — Correlation of GFP and mCherry fluorescence in tagged strains. (TIF) [file pgen.1004436.s016.tif]
